# The Mitochondrial Genomes of a Myxozoan Genus *Kudoa* Are Extremely Divergent in Metazoa

**DOI:** 10.1371/journal.pone.0132030

**Published:** 2015-07-06

**Authors:** Fumihiko Takeuchi, Tsuyoshi Sekizuka, Yumiko Ogasawara, Hiroshi Yokoyama, Ryoma Kamikawa, Yuji Inagaki, Tomoyoshi Nozaki, Yoshiko Sugita-Konishi, Takahiro Ohnishi, Makoto Kuroda

**Affiliations:** 1 Pathogen Genomics Center, National Institute of Infectious Diseases, Shinjuku-ku, Tokyo, Japan; 2 Department of Aquatic Bioscience, Graduate School of Agricultural and Life Sciences, The University of Tokyo, Bunkyo-ku, Tokyo, Japan; 3 Graduate School of Human and Environmental Studies, Graduate School of Global Environmental Studies, Kyoto University, Sakyou-ku, Kyoto, Japan; 4 Graduate School of Life and Environmental Sciences, University of Tsukuba, Tsukuba, Ibaraki, Japan; 5 Department of Parasitology, National Institute of Infectious Diseases, Shinjuku-ku, Tokyo, Japan; 6 Department of Food and Life Science, Azabu University, Sagamihara, Kanagawa, Japan; 7 Division of Microbiology, National Institute of Health Sciences, Setagaya-ku, Tokyo, Japan; Saint Mary's University, CANADA

## Abstract

The Myxozoa are oligo-cellular parasites with alternate hosts—fish and annelid worms—and some myxozoan species harm farmed fish. The phylum Myxozoa, comprising 2,100 species, was difficult to position in the tree of life, due to its fast evolutionary rate. Recent phylogenomic studies utilizing an extensive number of nuclear-encoded genes have confirmed that Myxozoans belong to Cnidaria. Nevertheless, the evolution of parasitism and extreme body simplification in Myxozoa is not well understood, and no myxozoan mitochondrial DNA sequence has been reported to date. To further elucidate the evolution of Myxozoa, we sequenced the mitochondrial genomes of the myxozoan species *Kudoa septempunctata*, *K*. *hexapunctata* and *K*. *iwatai* and compared them with those of other metazoans. The *Kudoa* mitochondrial genomes code for ribosomal RNAs, transfer RNAs, eight proteins for oxidative phosphorylation and three proteins of unknown function, and they are among the metazoan mitochondrial genomes coding the fewest proteins. The mitochondrial-encoded proteins were extremely divergent, exhibiting the fastest evolutionary rate in Metazoa. Nevertheless, the dN/dS ratios of the protein genes in genus *Kudoa* were approximately 0.1 and similar to other cnidarians, indicating that the genes are under negative selection. Despite the divergent genetic content, active oxidative phosphorylation was indicated by the transcriptome, metabolism and structure of mitochondria in *K*. *septempunctata*. As possible causes, we attributed the divergence to the population genetic characteristics shared between the two most divergent clades, Ctenophora and Myxozoa, and to the parasitic lifestyle of Myxozoa. The fast-evolving, functional mitochondria of the genus *Kudoa* expanded our understanding of metazoan mitochondrial evolution.

## Introduction

Myxozoans are 10–100-μm oligo-cellular parasites with two life cycle phases, the myxospore and actinospore phase. Most myxospores harmlessly parasitize fishes, yet myxospores of some species cause diseases in salmon, trout and whiting and thereby affect fisheries [1,2]. Recently, myxospores of one species were demonstrated to be the causal agent of food poisoning during consumption of raw flounder [3]. On the other hand, actinospores parasitize annelids, which are the definitive hosts of myxozoans [4].

The evolutionary positioning of Myxozoa based on the phylogeny of nuclear ribosomal RNA (rRNA) and a limited number of housekeeping genes was contradictory due to the fast evolutionary rate of Myxozoa [5–7]. The phylogeny of nuclear rRNA genes placed Myxozoa within Bilateria, the two-sided animals, including chordates, worms and mollusks [5]. Alternatively, the phylogeny of some housekeeping genes [6] and the similarity of myxozoan spores with stinging cells [8] suggested that this group belongs to Cnidaria, which are radially symmetric animals, including hydrozoans, jellyfishes and sea anemones. Phylogenomic studies utilizing an extensive number of nuclear-encoded genes may be effective in reconstructing evolutionary history and indeed could confirm that Myxozoa belong to Cnidaria [9,10].

The mitochondrion is an organelle that has its own genome, which evolved in parallel with the nuclear genome. Whereas mitochondrial genomes vary widely in size and gene content across eukaryotes, the metazoan mitochondrial genomes code an almost fixed set of proteins: 13 proteins for oxidative phosphorylation plus or minus a few [11,12]. Nevertheless, these mitochondrial genomes vary widely in evolutionary rate [13,14]. The metazoan mitochondria also vary in metabolism: for example, parasitic worms including *Fasciola hepatica* (liver fluke) and *Ascaris* (giant roundworm) perform fermentative metabolism under hypoxia utilizing the same set of mitochondrial enzymes as aerobic animals [15]. Hypoxia also changes the mitochondrial cristae structure in animals and yeast [16].

Despite the phylogenomic analyses of nuclear-encoded genes that place Myxozoa in Cnidaria, the evolution of parasitism and extreme body simplification in Myxozoa is not well understood, and no myxozoan mitochondrial DNA sequence has been reported to date. To elucidate the mitochondrial evolution of Myxozoa, we sequenced the mitochondrial genomes of the myxozoan species *Kudoa septempunctata*, *K*. *hexapunctata* and *K*. *iwatai* and compared them with other metazoans. To understand the evolution of mitochondrial and nuclear genomes as a whole, we also performed phylogenomic analysis by sequencing the transcriptome in *K*. *septempunctata*. In addition, to understand the role of mitochondria in the parasitic state of *K*. *septempunctata* myxospores, we observed the metabolism and structure. The current study can elucidate how the mitochondria changed while Myxozoa underwent the most extreme degenerative evolution observed in Metazoa.

## Results

**Fig 1:**
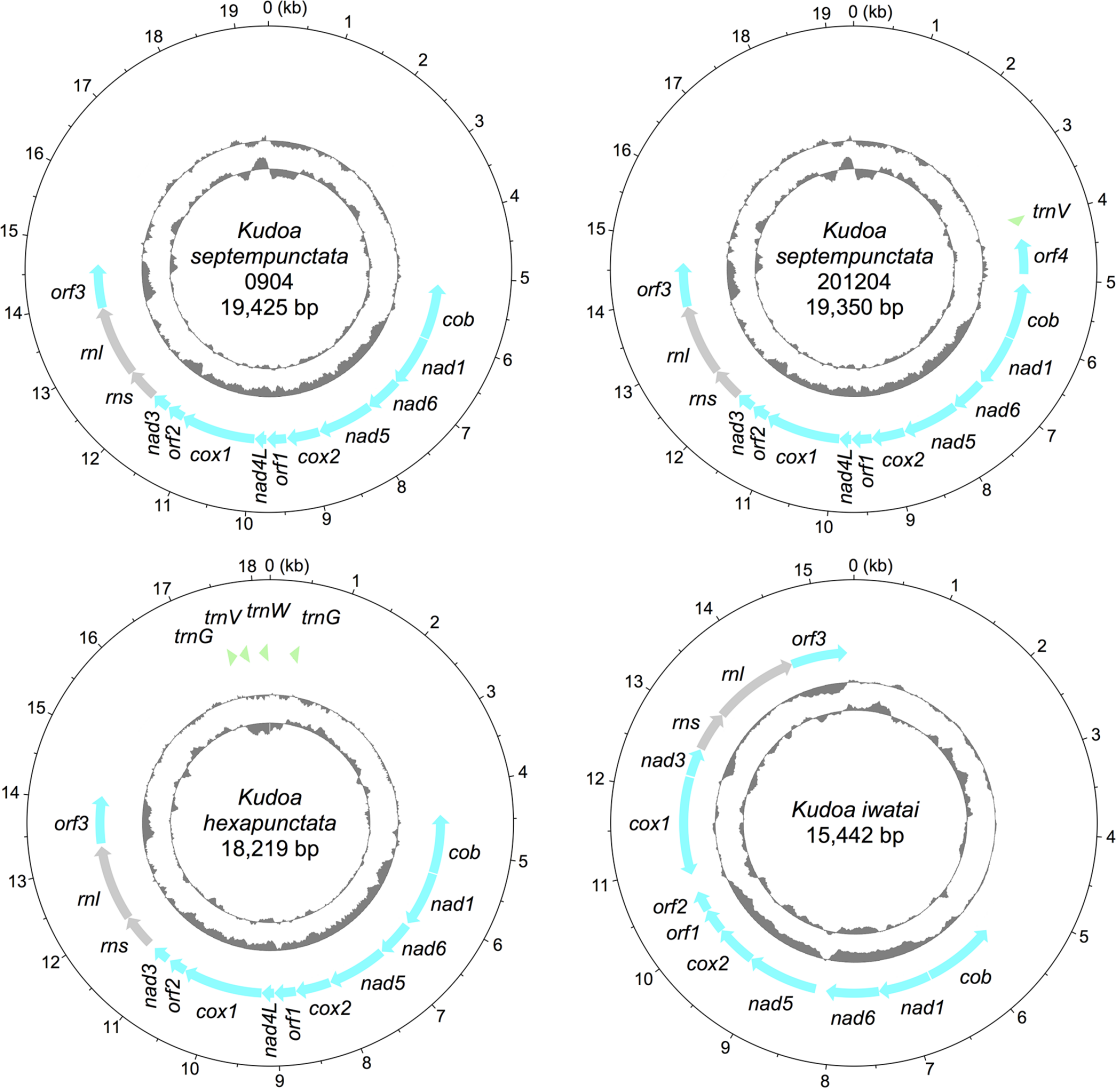
Mitochondrial genome maps of *Kudoa* species. Outer circle: protein-coding genes are represented by blue arrows, rRNAs by gray arrows and tRNAs by green arrowheads. Middle circle: plot of AT skew with the positive value towards the outside. Inner circle: plot of GC-content with higher %GC towards the outside.

We sequenced the mitochondrial DNA of *Kudoa* species using Illumina next-generation sequencers and confirmed the sequences with PCR experiments and PacBio long reads. The mitochondrial genomes were circular and contained approximately 15 to 19 kilobase pairs with GC-content 31% for *K*. *iwatai* and 42–44% for the others (Fig 1). By Southern blotting, we verified that the DNA sequence was not derived from nuclear chromosomes or from fish mitochondria (S1 Fig). The circular nature of mitochondrial DNA was confirmed by PCR experiments and by mapping PacBio long reads (S2 Fig). We also performed RNA-seq using total RNA and mapped the reads to the mitochondrial genome. The large- and small-subunit mitochondrial rRNA genes were clearly distinguishable by their abundant expression (S3 Fig). A few candidate transfer RNA (tRNA) genes were detected. Eight typical mitochondrial-encoded protein genes existed: cytochrome *c* oxidase subunits I, II (*cox1–2*), cytochrome *b* (*cob*), NADH dehydrogenase subunits 1, 3, 4L, 5–6 (*nad1*, *nad3*, *nad4L*, *nad5–6*). The genes were common in the *Kudoa* species, except for *nad4L*, which was undetected in *K*. *iwatai*. In addition, there were three genes with unknown function conserved in *Kudoa* species (*orf1–3*) and one unique to *K*. *septempunctata* isolate 201204 (*orf4*). The largest one, *orf3*, coded a membrane protein with around seven transmembrane regions. As is visible in the AT skew plot, adenine was less abundant than thymine on the strand coding the proteins. In all genomes, the genes were condensed in one half, and the remaining half (6–10 kb) coded almost no genes (only a few candidate tRNA genes in some isolates) and was not conserved among the *Kudoa* species.

**Fig 2:**
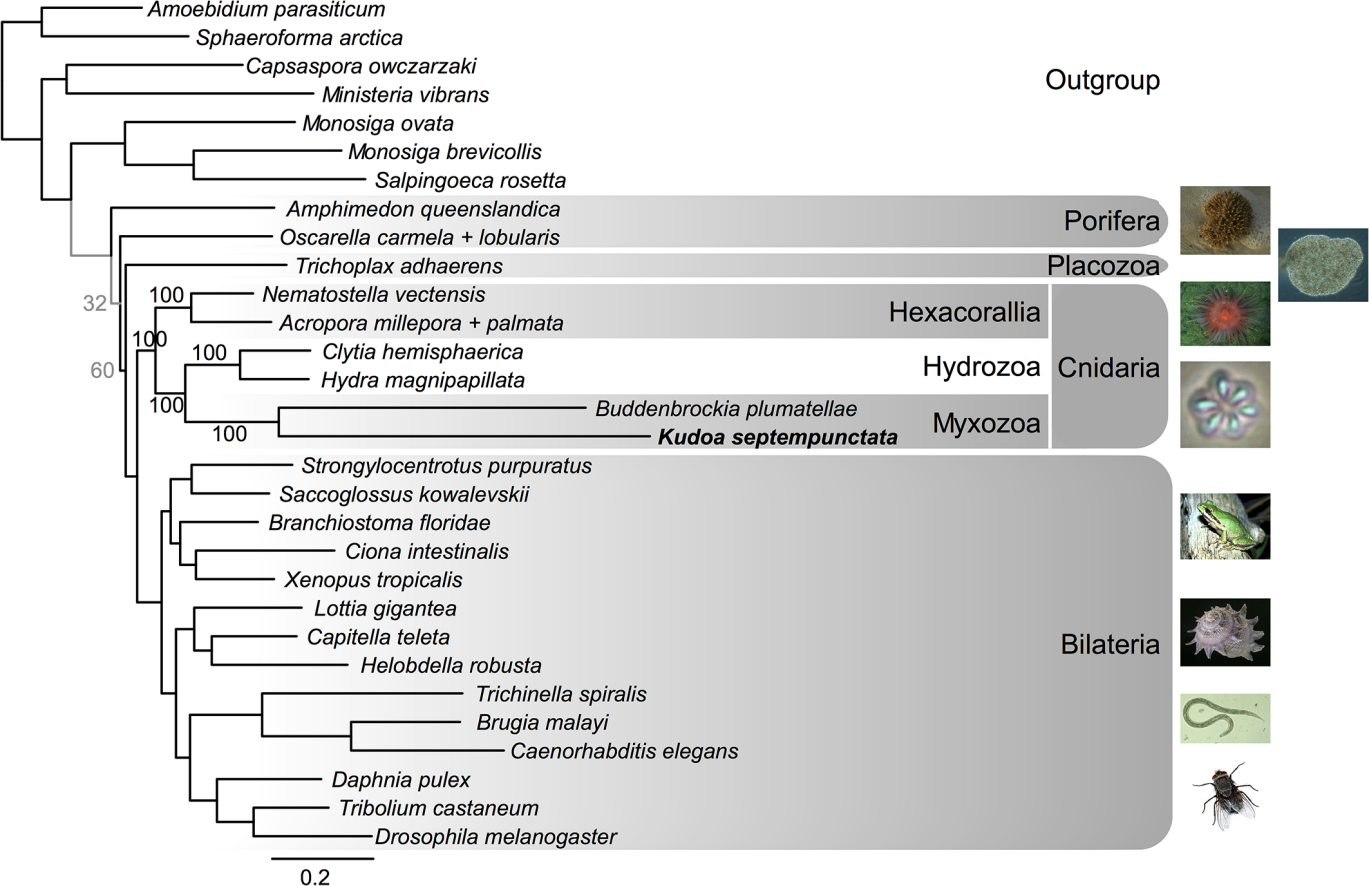
The phylogenetic tree of nuclear-encoded proteins. The trees were inferred using the maximum-likelihood method and bootstrapped 100 times. Branches with bootstrap support ≥80% are colored black, and those with support <80% are colored gray with the support value denoted. Support values are denoted also for the cnidarian clade. Image of *T*. *adhaerens* taken from [18], and others from the public domain.

**Fig 3:**
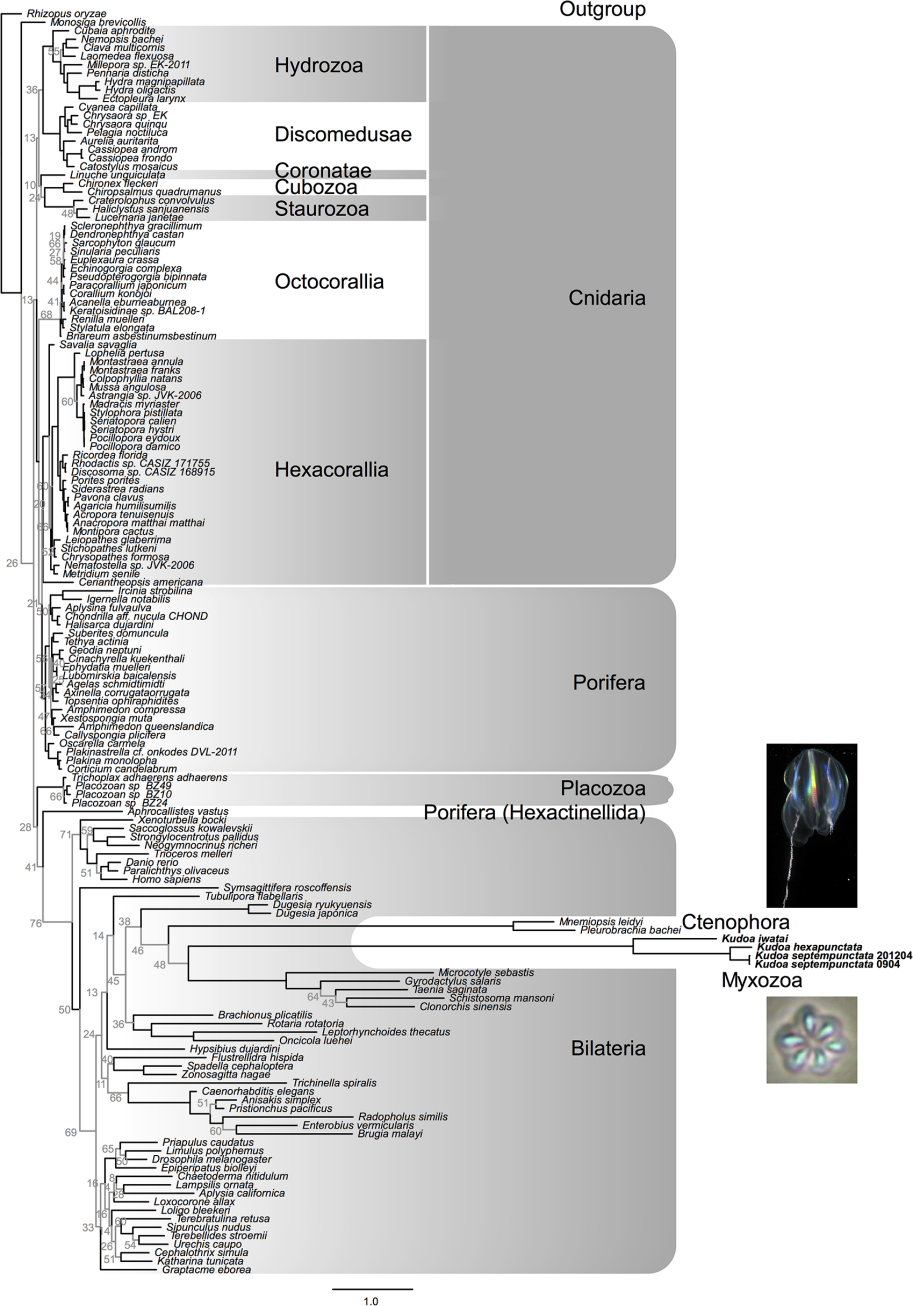
The phylogenetic tree of mitochondrial-encoded proteins. The trees were inferred using the maximum-likelihood method and bootstrapped 100 times. Branches with bootstrap support ≥80% are colored black, and those with support <80% are colored gray with the support value denoted.

**Fig 4:**
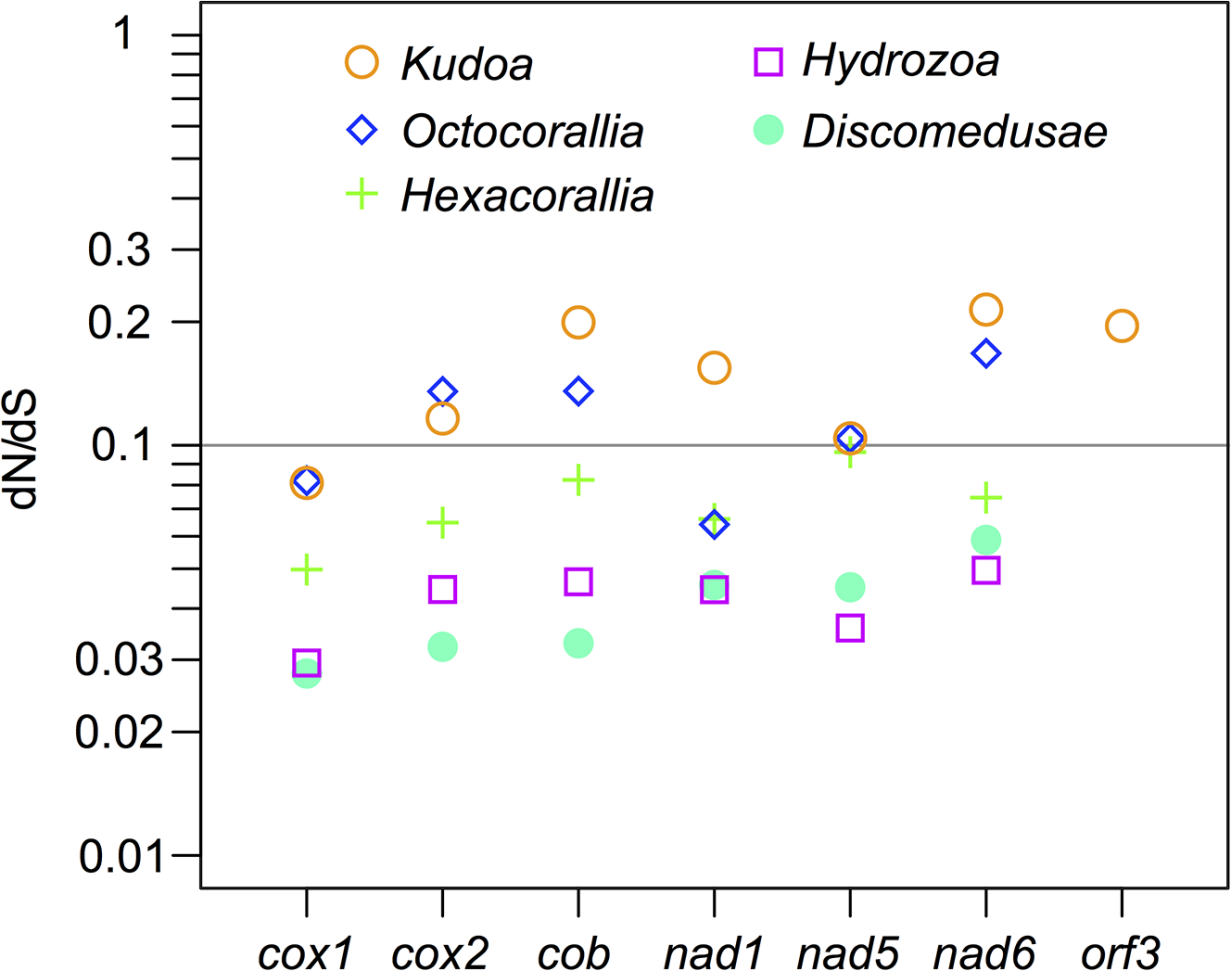
The relative rates of nonsynonymous and synonymous substitutions in mitochondrial-encoded protein genes, compared among the genus *Kudoa* and other cnidarian classes.

We next studied the phylogeny of mitochondrial-encoded and nuclear-encoded proteins. The phylogenetic tree of nuclear-encoded proteins (Fig 2) supported the previous phylogenomic studies [6,9,10] by suggesting the monophyly of the myxozoans, *Kudoa* and *Buddenbrockia*, and the position of myxozoans within Cnidaria with 100% bootstrap support. The evolutionary rate of myxozoans was faster than other metazoans, as visible from the longer branch. In the phylogenetic tree of mitochondrial-encoded proteins (Fig 3), the evolutionary rate was even faster for *Kudoa* and was faster than the previously known extreme case of ctenophores [13,14]. Because both *Kudoa* and *Ctenophora* have long branches, their branches were very likely falsely joined together with the long branches of *Platyhelminthes* (such as *Schistosoma mansoni*) due to long-branch attraction. In long-branch attraction, divergent species are known to be falsely grouped within a phylogenetic tree, while their branch lengths are unchanged [17]. There was no conclusive support on the deeper branches of the mitochondrial phylogenetic tree. To investigate if the fast mitochondrial evolutionary rate of *Kudoa* species could be due to positive selection or a lack of selective pressure (as in pseudogenes), we measured the relative rates of nonsynonymous and synonymous substitutions. The dN/dS ratios of mitochondrial-encoded protein genes in the genus *Kudoa* were approximately 0.1 and not remarkably higher than other cnidarians, suggesting that the genes are under negative selection to a similar degree in *Kudoa* and other cnidarian classes (Fig 4).

**Fig 5:**
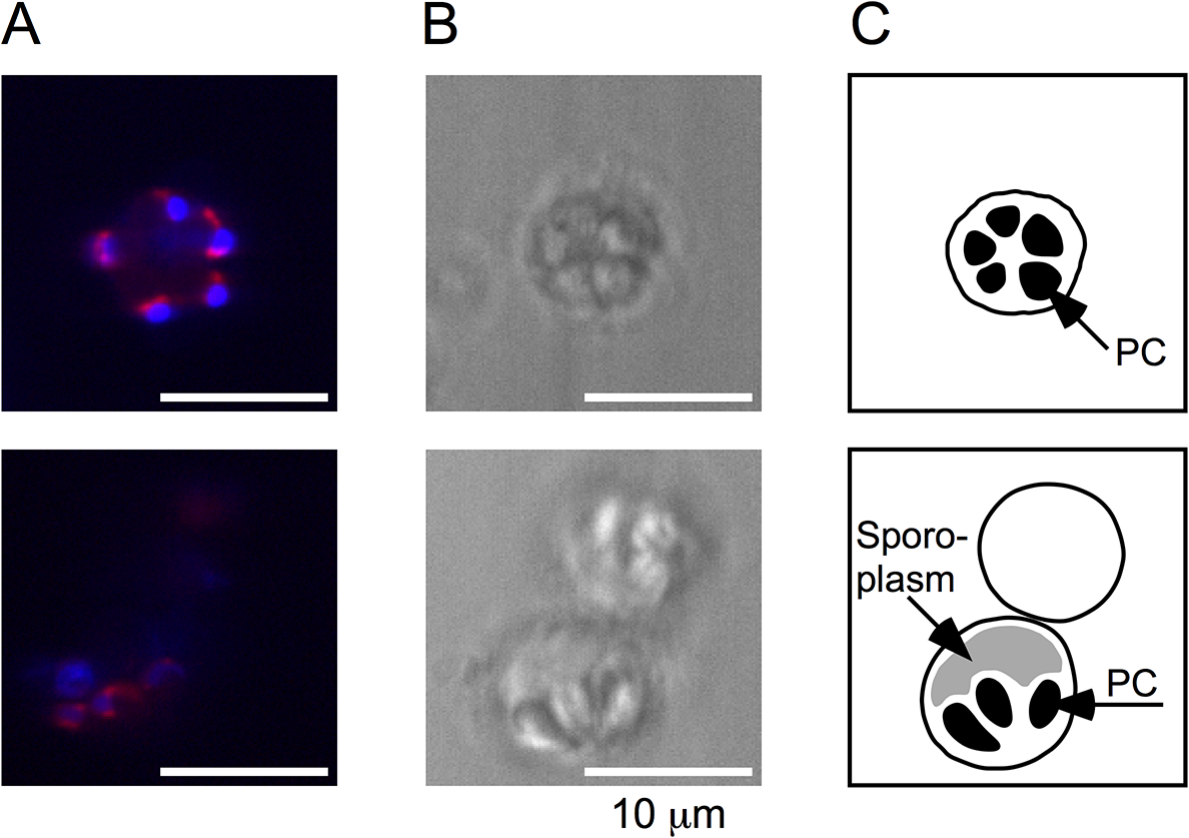
Mitochondrial aerobic respiration observed in *K*. *septempunctata* myxospores. (*A*) Confocal microscope images. Mitochondria are stained with Rhodamine 123 (red), and nuclei are stained with Hoechst 33258 (blue). (*B*) Bright field images. (*C*) Schematic representation of the myxospores. Polar capsules (PC) are arranged in a garlic shape within a myxospore. Within the sporoplasm, Rhodamine 123 accumulated beside the PCs, which indicates the negative electric potential in the mitochondrial membrane and suggests aerobic respiration. The myxospore in the top panel appears in transverse section, and the myxospore in the lower half of the bottom panel appears in radial section. Images of other sections are shown in S4 Fig.

**Fig 6:**
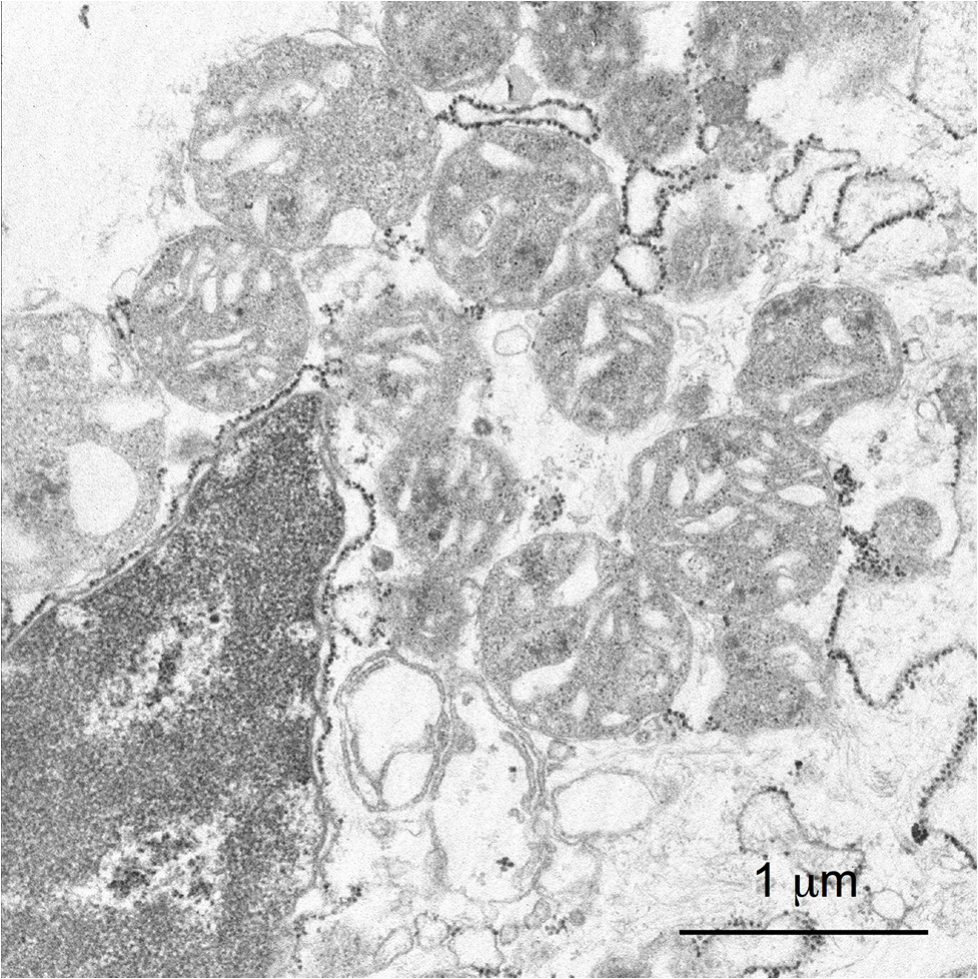
Transmission electron microscopy of mitochondria in sporoplasm released from *K*. *septempunctata* myxospores.

To examine if oxidative phosphorylation is maintained under the extremely divergent mitochondrial genome, we analyzed the transcriptome, metabolism and structure of mitochondria in *K*. *septempunctata*. We used *K*. *septempunctata* myxospores, which could be purified fresh from an aquaculture-raised flounder [3]. In the transcriptome, all genes of the citrate cycle and around half of the genes of each complex for oxidative phosphorylation were expressed (S1 Table). Additionally, we observed *in vivo* the aerobic respiration in *K*. *septempunctata*. In typical mitochondria, the first four complexes of oxidative phosphorylation pump protons out of the mitochondrial inner membrane, and the last complex uses the proton gradient to synthesize ATP. We stained *K*. *septempunctata* myxospores with Rhodamine 123, which detects proton gradients in the mitochondria. Rhodamine 123 accumulated next to the polar capsules of the myxospores, suggesting that the mitochondria therein were performing aerobic respiration (Fig 5, S4 Fig). Furthermore, by using transmission electron microscopy (Fig 6), we observed the tubular cristae structure in the mitochondria, as in other myxozoans [4]. The diameter of a mitochondrion was 1 μm in both the confocal and electron microscopy images. The transcriptome and microscopy data suggest active aerobic respiration in *K*. *septempunctata* mitochondria.

## Discussion

The mitochondrial genomes of the *Kudoa* species—the first sequenced in Myxozoa—were extremely divergent from other metazoans. Nevertheless, active oxidative phosphorylation in *K*. *septempunctata* was suggested by the proton gradient and cristae structure of the mitochondrial membrane, the expression of genes for oxidative phosphorylation and negative selection imposed on mitochondrial-encoded protein genes.

### Mitochondrial genomes

The *Kudoa* mitochondrial genomes code for rRNAs, tRNAs and 11 proteins (Fig 1). Few tRNAs were encoded in the mitochondrial genome, and the remaining are likely transported from the cytosol to the mitochondria, as in the case of some metazoans [11]. Metazoan mitochondrial genomes mostly code 13 proteins for oxidative phosphorylation, among which *cox3*, *nad2*, *nad4*, *atp6* and *atp8* were unidentified in *Kudoa* species. Eleven proteins were the least encoded in metazoan mitochondrial genomes (http://www.ncbi.nlm.nih.gov/genomes/OrganelleResource.cgi?taxid=33208), which have been found in Ctenophora [13,14], Chaetognatha [19–21] and a species of tree frog [22].

### Phylogenetic analysis

The phylogenomic analysis of *K*. *septempunctata* nuclear-encoded proteins firmly placed Myxozoa in Cnidaria (Fig 2), in accordance with previous studies [6,9,10]. While the previous studies utilized orthologous proteins, we used a dataset of protein domains that appear in single copies in most of the genomes, which share less than 10% with conventional phylogenomic datasets [23]. Thus, our data strengthens the placement of Cnidaria on the basis of the newly sequenced taxon as well as the complementary genetic sites. In general, phylogenetic analysis of mitochondrial-encoded proteins is informative, owing to abundantly available taxa and clearly orthologous genes [11], and has complemented the phylogenetic analysis of nuclear rRNA genes [24]. However, for Myxozoa and Ctenophora, the fast evolutionary rate was clearly demonstrated, and the evolutionary history could not be reconstructed (Fig 3).

### Oxidative phosphorylation

The activity of mitochondrial oxidative phosphorylation in *K*. *septempunctata* myxospores was suggested by metabolic and structural observations in addition to genomic and transcriptomic data. The proton gradient (Fig 5) and cristae structure (Fig 6) of the mitochondrial membrane were not essentially different from those of other aerobic animals. However, measuring the activity of each complex for oxidative phosphorylation is necessary for the definitive understanding of cellular respiration.

### Fast evolutionary rate

The mitochondrial-encoded protein genes in the *Kudoa* species showed an evolutionary rate that was much faster than other metazoans, but they were under negative selection (Figs 3 and 4). The high dN (nonsynonymous substitution rate) and dN/dS ≈ 0.1 indicates high dS (synonymous substitution rate). This suggests the acceleration of the mitochondrial nucleotide evolutionary rate in Myxozoa. A fast mitochondrial evolutionary rate is known for Ctenophora, which is the oldest lineage of Metazoa and has evolved a neural system in parallel with the Bilateria+Cnidaria clade [13,14,25,26]. Although Ctenophora and Myxozoa are taxonomically unrelated and differ in body plan and life cycle, the population genetic characteristics proposed to account for the fast evolutionary rate in Ctenophora [13] are shared by Myxozoa. There are two characteristics that cause a small effective population size, which in turn causes a faster evolutionary rate [27]. First, ctenophores are capable of massive reproduction followed by bottlenecks when the seawater is nutritionally rich [28]. In the case of myxozoans, massive reproduction and bottleneck occur by parasitism [29]; myxozoans can reproduce massively in a host (either fish or annelid), but once released to the water, a very small proportion reaches the next host. Secondly, ctenophores are capable of self-fertilization, which causes inbreeding. Inbreeding directly increases the evolutionary rate of nuclear-encoded (mitochondrial) genes by reducing effective population size and indirectly increases the rate of mitochondrial-encoded genes [13]. In Myxozoa, the sexual stage occurs in annelids. Mating would usually occur between descendant cells of one actinospore that intruded to an annelid, which essentially is self-fertilization [30].

As an alternative explanation for accelerated evolution, we speculate that positive selection due to the parasitic lifestyle acted on ancestral Myxozoa. Although the mitochondrial dN/dS ratio within the genus *Kudoa* did not differ from other cnidarians, the ratio in the ancestor of *Kudoa* is unknown. Compared to other Cnidarians, Myxozoans have a completely different niche that poses particular environmental pressure: the intracellular parasitism to alternate hosts requires the abilities to invade hosts and evade host immunity [4,31]. There are non-metazoan parasites with similar life cycles that underwent unique mitochondrial evolution. For example, malaria parasites parasitize humans and mosquitos and have a mitochondrial genome that only encodes ribosomal RNAs and three proteins [32]. Additionally, *Entamoeba histolytica* is an anaerobic human parasite that has no DNA in its mitochondrion-related organelle [33]. Considering the divergent genome of *Kudoa*, Myxozoa could have evolved capabilities to adjust to the environment inside the host cells, which is in parallel with other unrelated parasites living in a similar environment. However, parasitism does not always imply an unusual mitochondrial genome, as in the cases of “standard” metazoan mitochondrial genomes in parasitic nematodes and flatworms (Fig 3). Our work suggests that the mitochondrial genome of Myxozoa is potentially divergent in general, yet it must be confirmed by sequencing more species in the future.

## Materials and Methods

### Preparation and sequencing of DNA and RNA

Total DNA and RNA of *K*. *septempunctata* were extracted from *Paralichthys olivaceus* muscle parasitized with *K*. *septempunctata* myxospores (isolate 0904) or from purified myxospores (isolate 201204) using the RecoverAll Total Nucleic Acid isolation kit (Ambion), as previously reported [3]. Total DNA of *K*. *hexapunctata* (isolate 2012.6.3) and *K*. *iwatai* (isolate KI-001) were extracted from myxospores purified from *Thunnus orientalis* and *Acanthopagrus latus*, respectively. DNA and RNA were sequenced on Illumina next-generation sequencers and a PacBio single molecule sequencer (S2 Table). DNA libraries were prepared using the Paired End Genomic DNA Sample Prep Kit (Illumina) or Nextera DNA Sample Prep Kit (Epicenter, Madison, WI) and sequenced on a Genome Analyzer IIx system or MiSeq system (Illumina). For *K*. *septempunctata* isolate 0904, another DNA library was prepared using the SMRTBell Template Prep Kit 1.0 and sequenced on a PacBio RSII sequencer (Pacific Biosciences). For a few regions with low Illumina read depth, we performed PCR and Sanger sequencing of the DNA using the BigDye Terminator v3.1 Cycle Sequencing Kit on a 3730xl DNA Analyzer (Life Technologies). Complementary DNA (cDNA) libraries for RNA were prepared using the ScriptSeq mRNA-Seq Library Preparation Kit (Epicentre) and sequenced on a Genome Analyzer IIx system. The myxozoans and fishes used in the current study do not belong to mammals, birds and reptiles and thus are not regulated by the Act on Welfare and Management of Animals (Japanese law 1973–105). For the same reason, permission for the experiments in the study was waived according to the rules of the IACUC of the National Institute of Infectious Diseases. The fishes were caught by fishermen who held licenses for commercial fishing and were processed to meat under ordinary procedures with minimum pain. There were no invasive procedures. Samples for the study were picked out from the meat of deceased fish; for each isolate of *Kudoa*, at most 10 grams of muscle beneath the lateral line was sampled from one fish. The fishes were killed for dietary use and not for this study. The three fish species were not considered to be threatened or endangered then according to the IUCN Red List.

### Quality trimming and assembly of DNA reads

Mitochondrial genome sequences were assembled from Illumina and Sanger reads; PacBio reads were used to confirm the *K*. *septempunctata* isolate 0904 assembly. We omitted any Illumina read pair that included an adapter sequence using the SeqPrep program (released June 1, 2013, https://github.com/jstjohn/SeqPrep). The first and last bases of a read, as well as low quality bases, were trimmed using the PoPoolation software (version 1.2.2) [34]. We retained reads of length ≥40 bp and with no ‘N’ bases.

The Illumina reads were assembled using the ABySS software (version 1.3.6) [35] or the Platanus software (version 1.2.1) [36]. A few sequence gaps were closed by PCR followed by Sanger sequencing. Contig sequences were refined by repeating the process of mapping the reads and taking the consensus sequence. Mapping was performed using the BWA (version 0.6.1) [37] and SAMtools (version 0.1.18) [38] software and visualized using the IGV (version 2.0.34) [39] and GenomeJack (version 2.1, Mitsubishi Space Software) software. The mapping to a circular contig was performed by cutting a linear contig at different sites.

Mitochondrial genome contigs were identified by the existence of *cox1* gene and had distinguishably higher read depths than other contigs. The depth (or redundancy) of the sequenced reads reflects the amount of the source DNA. For *K*. *septempunctata* isolate 0904, the read depth was ~27,000 for the mitochondrial genome, ~100 for the nuclear genome and lower for fish and other contaminant DNA (S5 Fig).

The assemblies were confirmed by overlapping PCR experiments that covered the mitochondrial genomes (S3 Table). We used the PrimeSTAR GXL DNA polymerase (TaKaRa) under the following PCR conditions: primary denaturation at 95°C for 3 min; 30 cycles of denaturation at 95°C for 30 seconds, annealing at 55°C for 30 seconds and extension at 68°C for 1 min per kilobase; final extension at 68°C for 5 min. The *K*. *septempunctata* isolate 0904 assembly was confirmed also by mapping PacBio long reads using the BLASR software (released June 11, 2014) [40] (S2 Fig).

### Southern blotting

To verify that the obtained genome sequence was derived from the mitochondrial chromosome and not from the nuclear copies of mitochondrial DNA (in nuclear chromosomes) or from fish mitochondria, we performed Southern blotting. We performed pulsed field electrophoresis of *K*. *septempunctata* isolate 0904 total DNA and *Kudoa*-free *Paralichthys olivaceus* total DNA (control) on a 1% agarose gel in 0.5x TBE. Using CHEF-DR II system (Bio-Rad), the electrophoresis was performed under 6 V/cm, 120° angle in two blocks: the run time was 3 h with 0.1–1 sec switch time ramp, followed by 11 h with 1–2 sec switch time ramp. DNA was transferred from the gel to a GeneScreen Plus Hybridization Transfer Membrane (PerkinElmer) by the alkaline transfer method. DNA probes were labeled using Amersham Gene Images AlkPhos Direct Labelling and Detection System (GE Healthcare) and hybridized to the DNA on the membrane. The probes were PCR products amplifying the region at 10194–11194 bp (within *cox1* gene) or the region at 17170–17760 bp (no gene) of *K*. *septempunctata* isolate 0904 genome.

### Quality trimming, mapping and assembly of RNA reads

Adaptor removal and quality trimming of RNA reads were performed similarly as done for the DNA reads, with an alternation of retaining reads of length ≥25 bp (S2 Table). Mapping of RNA reads to the mitochondrial genomes was performed as described above for the mapping of DNA reads. Transcriptome sequences of *K*. *septempunctata* isolate 201204 were *de novo* assembled from RNA reads using Trinity software (released Feb 25, 2013) [41].

### Annotation of ribosomal RNA genes

For the two isolates of *K*. *septempunctata*, the large and small subunit mitochondrial ribosomal RNA (rRNA) genes were clearly distinguishable in the genome as a region where abundant RNA reads were mapped (S3 Fig). Although the two genes were located beside each other in the genome, a valley in the mapping depth could demarcate the two. The sequence similarity of the two gene sequences with the homologous genes in other metazoans was verified by multiple alignment using the MAFFT program (version 6.864b) [42] with the Q-INS-i option. The rRNA genes in the other two *Kudoa* species were located by aligning the *K*. *septempunctata* rRNA genes and assuming the same gene size.

### Annotation of transfer RNA genes

A genome-wide search for transfer RNA (tRNA) genes was performed using the following methods: 1) DOGMA annotator [43] with Cove cutoff score 7; 2) BLAST search against Genomic tRNA Database (http://gtrnadb.ucsc.edu) with E-value <1; 3) Rfam database search [44]; 4) tRNAscan-SE software (version 2.1) [45] with Cove cutoff score 1; 5) Split-tRNA-Search (http://www.prodoric.de/sts/); 6) SPLIITS software (version 1.1) [46]; 7) SPLITSX software (version 1) [47]. Methods 1, 4–7 allow for introns and/or split tRNA. The hits were screened by visually inspecting the stems and loops that comprised the cloverleaf form secondary structure. The hits that overlapped with protein-coding or rRNA genes were omitted. The candidates that remained were all discovered by the DOGMA annotator (S6 Fig).

### Annotation of protein-coding genes

To identify protein-coding genes, we extracted all possible protein sequences comprising ≥200 amino acids or of ≥50 amino acids and homologous to sequences in other species. Specifically, we translated the mitochondrial DNA sequences under six frames (using the NCBI translation table 4 for cnidarian mitochondria), extracted all possible protein sequences of length ≥50 and queried each protein sequence against the NCBI non-redundant protein database (April 18, 2014 release) and against extracted sequences of other *Kudoa* species using the BLASTP program (version 2.2.25; option-W 2) [48]. A gene product was required to have more than one hit with E-value <0.001. A gene product comprising ≥200 amino acids was adopted regardless of hits. We found five known genes—*cox1*, *cox2*, *cob*, *nad1* and *nad5*—four genes unknown but conserved in *Kudoa* species, and one gene unique to *K*. *septempunctata* isolate 201204.

We also performed a relaxed search allowing introns, but did not detect additional genes. Specifically, the amino acid sequences of 13 typical mitochondrial-encoded genes (*atp6*, *atp8*, *cox1–3*, *cob*, *nad1–6*, *nad4L*) for the cnidarian species listed in [49] were searched against the nucleotide sequences of *Kudoa* mitochondria, using the exonerate program [50] (version 2.2.0; option—model protein2genome—geneticcode 4—exhaustive y). We also searched for conserved coding sequences between the nucleotide sequences of *Kudoa* species (option—model coding2coding—geneticcode 4—exhaustive y). Only hits with alignment scores ≥80 were retained.

Furthermore, to detect less conserved genes, we searched the mitochondrial genomes by using hidden Markov models (HMMs) for the typical mitochondrial-encoded proteins (listed above). We either searched the Pfam HMM [51] against the nucleotide sequences using the GeneWise program (version 2.4.1, option-codon table_4-both-trans-hmmer, score >15) [52] or searched the Pfam HMM against translated sequences of ≥50 amino acids using the hmmsearch program (version 3.0, E-value <0.001) [53]. Among the unknown conserved genes found by BLASTP search, one was identified as *nad6* by using the HMM search. In addition, *nad3* and *nad4L* were newly discovered.

In the above gene-finding step, homology search was performed for the tentative coding sequence (CDS) defined as the longest possible between stop codons in a specific frame. Some of the resulting CDSs were overlapping at termini. To resolve the overlap, the beginning of a CDS was trimmed to the position of the foremost initiation codon (of translation table 4) that did not overlap with other CDSs. For the five genes with BLASTP homology to know genes, global alignment was verified by visually inspecting BLASTP homology plots between *Kudoa* and other species. For genes of unknown function, protein domains were predicted using the InterPro Scan software (version 5.7) [54]. Gene annotation was visualized using the Artemis [55], DNAPlotter [56] and GView [57] software.

### Functional annotation of the nuclear transcripts

To perform phylogenomic analysis and to investigate the mitochondrial metabolism in *Kudoa*, we functionally annotated the transcriptome of *K*. *septempunctata* isolate 201204. From the *de novo* assembly of the transcriptome (42,893 assembled transcripts), we first omitted transcripts derived from the host fish, *Paralichthys olivaceus*. For this, with each transcript as a query, homologous sequences were searched in the NCBI non-redundant nucleotide database (April 17, 2014 release) and non-redundant protein database (June 9, 2013 release) using BLASTN and RAPSearch2 (version 2.14, option-a T) [58] software, respectively. We omitted the transcripts assigned to *Teleostomi* (vertebrates) using MEGAN software (version 5.1.4, option MinScore = 0, TopPercent = 10, MaxExpected = 0.01, MinSupport = 1). Next, the remaining 40,028 transcripts were functionally annotated using the KEGG Automatic Annotation Server by searching for the single-directional best hit against the gene data set of predefined eukaryotes plus *Nematostella vectensis* and *Hydra vulgaris* [59]. The server also analyzed the presence or absence of KEGG pathway genes in the *K*. *septempunctata* transcriptome.

### Multiple alignments of mitochondrial-encoded proteins

For the phylogenetic analysis of mitochondrial-encoded proteins, *Kudoa* species were compared with 149 representative species of the Metazoa (see S4 Table). Cnidaria, Placozoa and Porifera were sampled extensively by including the species listed in [49]. We included at least one species from each phylum of Bilateria, as long as complete mitochondrial genome sequences were available. In addition, as outgroups for Metazoa, we chose the closest unicellular outgroup, *Monosiga brevicollis*, and a fungus, *Rhizopus oryzae*. To find taxa, we used the NCBI Taxonomy Browser (http://www.ncbi.nlm.nih.gov/Taxonomy/Browser/wwwtax.cgi).

To perform phylogenetic analysis, we first computed multiple alignments for each of the mitochondrial-encoded proteins—*cox1*–*2*, *cob*, *nad1* and *nad5*. We excluded the less conserved proteins, for which homology was undetectable by BLASTP search (see above). The protein sequences of *Kudoa* species were divergent from other species, which could flaw the quality of multiple sequence alignments. To obtain better alignment, we performed alignments guided by structure prediction (alpha helices, beta sheets, transmembrane regions) using the PRALINE program [60]. From the obtained alignment, we extracted stretches of continuous structures and discarded non-conserved regions in-between. For each of the extracted stretches, the alignment was refined using the MAFFT program with the L-INS-i option; *Kudoa* species and others were pre-aligned separately and combined under relaxed gap penalty (—addprofile—op 0.51—lop -0.67—lep 0.03—lexp -0.03) to cope with the sequence divergence. Alignment sites with a substantial proportion of gaps were removed using the trimAl program (version 1.2, option-gt 0.9) [61]. The trimmed alignments were then concatenated. When the concatenated sequences were identical in multiple species, one of them was retained. The final alignment included 1413 sites for 149 taxa.

### Multiple alignments of nuclear-encoded proteins

To compare the evolution of mitochondrial and nuclear genomes, we performed phylogenetic analysis of nuclear proteins. We used the phylogenomic analysis based on conserved protein domains that appear in single-copy in most of the genomes [9,10,23]. The 78 original untrimmed single protein data sets of metazoans and outgroups were kindly provided by Dr. Ruiz-Trillo (Universitat de Barcelona). We excluded fungi and other outgroup taxa and kept Holozoa lineages—Metazoa, Choanoflagellata (*Monosiga brevicollis*, *Monosiga ovate* and *Salpingoeca rosetta*), Ichthyosporea (*Amoebidium parasiticum* and *Sphaeroforma arctica*) and Filasterea (*Capsaspora owczarzaki* and *Ministeria vibrans*)—as we focused on the internal branching patterns of Metazoa. We added the transcriptome data for *K*. *septempunctata* isolate 201204 (described above) and another myxozoan, *Buddenbrockia plumatellae* (downloaded from DDBJ/EMBL/GenBank) [6]. We extracted protein sequences matching a domain from the transcriptome by protein homology search using the TBLASTN program with the criterion of E-value < 0.05. The myxozoan protein sequences were added to the aligned sequence of other species using the MAFFT program, and ambiguously aligned positions were excluded by visual inspection.

Each of the aligned single-protein data sets were subjected to maximum-likelihood (ML) phylogenetic analysis using the RAxML software (version 7.2.6) [62]. We used the LG model [63], incorporated empirical amino acid frequencies and approximated among-site rate variation by a discrete gamma distribution with four categories (LG+Gamma+F model). Heuristic tree searches were performed based on ten distinct parsimony starting trees, each generated by distinct random stepwise sequence addition. One hundred bootstrap replicates were generated for each data set and then subjected to ML analysis with the LG+Gamma+F model. In ML bootstrap analyses, heuristic tree searches were performed from a single parsimony tree estimated by random stepwise addition per replicate. All of the protein trees were free of clades falsely split with ≥70% bootstrap support, which indicated that none of the 78 data sets contained paralogous proteins or proteins suspiciously derived from lateral gene transfer. The final alignment included 23,668 positions for 30 taxa of Holozoa. The aligned data sets and the proportion of missing sites are available from https://sites.google.com/site/ryomakamikawa/Home/dataset.

### Phylogenetic analysis

The phylogenetic analysis was performed separately for the mitochondrial-encoded proteins and the nuclear proteins. We used the ML method implemented in the RAxML software. We applied the MTZOA+Gamma+F model for mitochondrial proteins and the LG+Gamma+F model for nuclear proteins and bootstrapped 100 times as described above. The MTZOA model specifies the empirical exchange rates of amino acids for known mitochondria of Metazoa [64]. Phylogenetic trees were visualized using the Archaeopteryx program (version 0.9891 beta) [65] and FigTree program (version 1.3.1, http://tree.bio.ed.ac.uk/software/figtree/).

### Selective pressure on mitochondrial-encoded protein genes

To estimate the selective pressure imposed on mitochondrial-encoded protein genes in *Kudoa* species, we computed the relative rates of nonsynonymous and synonymous substitutions and compared them with other cnidarian classes. We chose the classes with abundant sequenced mitochondrial genomes—*Hydrozoa*, *Discomedusae*, *Octocorallia* and *Hexacorallia* (see S4 Table)—and the longer protein genes—*cox1–2*, *cob*, *nad1*, *nad5–6 and orf3*. We collected proteins of a gene for each set of species (including cnidarians), computed the protein sequence alignment using the MAFFT L-INS-i program and then obtained the nucleotide sequence alignment using the PAL2NAL program (version 14) [66]. The maximum-likelihood estimate of dN/dS was computed using the CodeML program of the PAML package (version 4.8) [67].

### Observation of mitochondrial aerobic respiration

To determine whether the mitochondria of *K*. *septempunctata* were actively respiring, we used Rhodamine 123 (Sigma-Aldrich). Rhodamine 123 is a cationic fluorescent dye that distributes according to the negative membrane potential across the mitochondrial inner membrane [68]. Fresh *K*. *septempunctata* myxospores were collected from flounder obtained from a fish farm, as previously described [3]. Approximately 5×10^4^ spores were suspended in 50 μl of 10 μg/ml Rhodamine 123 in D-MEM buffer (Wako Pure Chemical Industries) and incubated for 15 min. The sample was washed three times by centrifugation at 1300 *g* for 5 min, the supernatant was removed, and the sample was resuspended in 50 μl of fresh D-MEM buffer. Finally, the sample was suspended in 50 μl of 10 μg/ml Hoechst 33258 (Dojindo Molecular Technologies) in D-MEM buffer for counterstaining of the nucleus. We used the confocal laser scanning microscope LSM 7 LIVE (Carl Zeiss) for observation. The experiment was performed at room temperature.

### Transmission electron microscopy of mitochondria

Fresh *K*. *septempunctata* myxospores were inoculated to human adenocarcinoma cell line Caco-2 cells and incubated at 37°C for 1 h. We observed the structure of mitochondria using a transmission electron microscope, as previously described [69].

## Supporting Information

S1 Fig Southern blot for the electrophoresis of *K*. *septempunctata* isolate 0904 total DNA and *Kudoa*-free fish DNA. (PDF)

S2 Fig Alignment of PacBio long reads to the mitochondrial genome of *K*. *septempunctata* isolate 0904. (PDF)

S3 Fig RNA reads mapped to the *K*. *septempunctata* isolate 0904 mitochondrial genome. (PDF)

S4 Fig Mitochondrial aerobic respiration observed in *K*. *septempunctata* myxospores. (PDF)

S5 Fig DNA sequencing depth of the mitochondrial chromosome of *K*. *septempunctata* isolate 0904. (PDF)

S6 Fig Transfer RNA genes discovered in the mitochondrial genomes of *Kudoa* species. (PDF)

S1 Table Mitochondrial metabolism genes expressed in *K*. *septempunctata* isolate 201204. (PDF)

S2 Table DNA and RNA library preparation and sequencing. (PDF)

S3 Table Primers used to confirm the mitochondrial genome assemblies. (PDF)

S4 Table Mitochondrial genomes used for phylogenetic analyses. (PDF)
