# Supplementary material for: The Mitochondrial Genomes of a Myxozoan Genus Kudoa Are Extremely Divergent in Metazoa
Source: PLoS One. 2015 Jul 6;10(7):e0132030. doi: 10.1371/journal.pone.0132030 (PMC4492933; doi:10.1371/journal.pone.0132030)

S1 Fig.

Southern blot for the electrophoresis of *K. septempunctata* isolate 0904 total DNA (in two lanes) and *Kudoa*-free fish DNA (control lane). In *Kudoa* lanes, two bands (blue arrowheads) are detected at ~23 and ~30 kb (measured by linear double-stranded DNA size marker; red lines) in the lanes for *Kudoa*, but no band is detected in the control lane. Thus, the obtained mitochondrial genome sequence does not derive from nuclear chromosomes or from fish mitochondria; under those denied cases, the bands should have appeared only at the wells of *Kudoa* lanes or in the control lane.

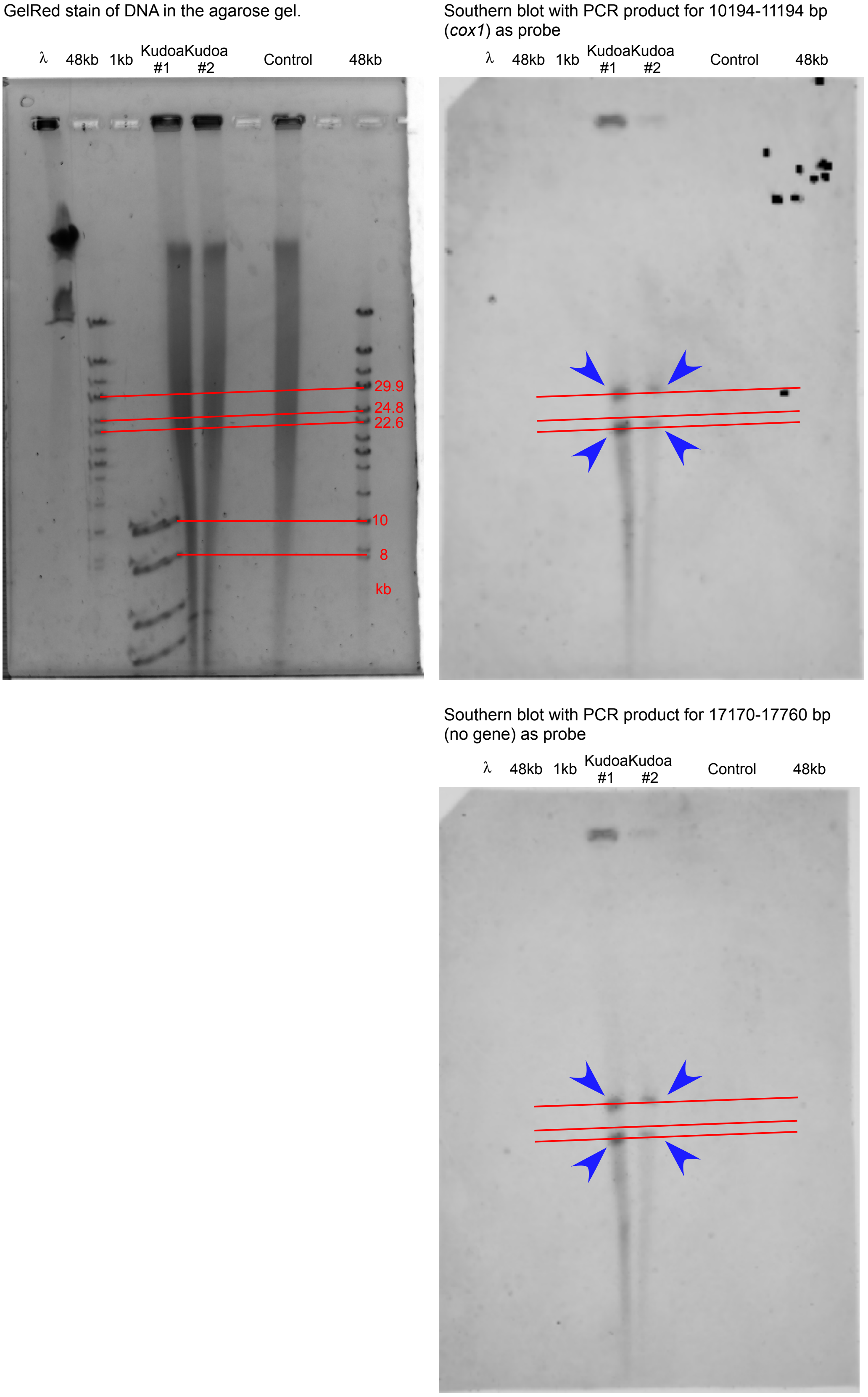

Supplement: S1 Fig — (PDF) [file pone.0132030.s001.pdf]
