# Supplementary material for: The Mitochondrial Genomes of a Myxozoan Genus Kudoa Are Extremely Divergent in Metazoa
Source: PLoS One. 2015 Jul 6;10(7):e0132030. doi: 10.1371/journal.pone.0132030 (PMC4492933; doi:10.1371/journal.pone.0132030)

S2 Fig.

Alignment of PacBio long reads to the mitochondrial genome of *K. septempunctata* isolate 0904. The top 100 reads with the highest mapping scores are shown. Each horizontal bar represents a PacBio read aligned to the genome at the position indicated in the horizontal axis. The five reads in the bottom (blue) are split to the two ends, because they cross the origin of the circular genome.

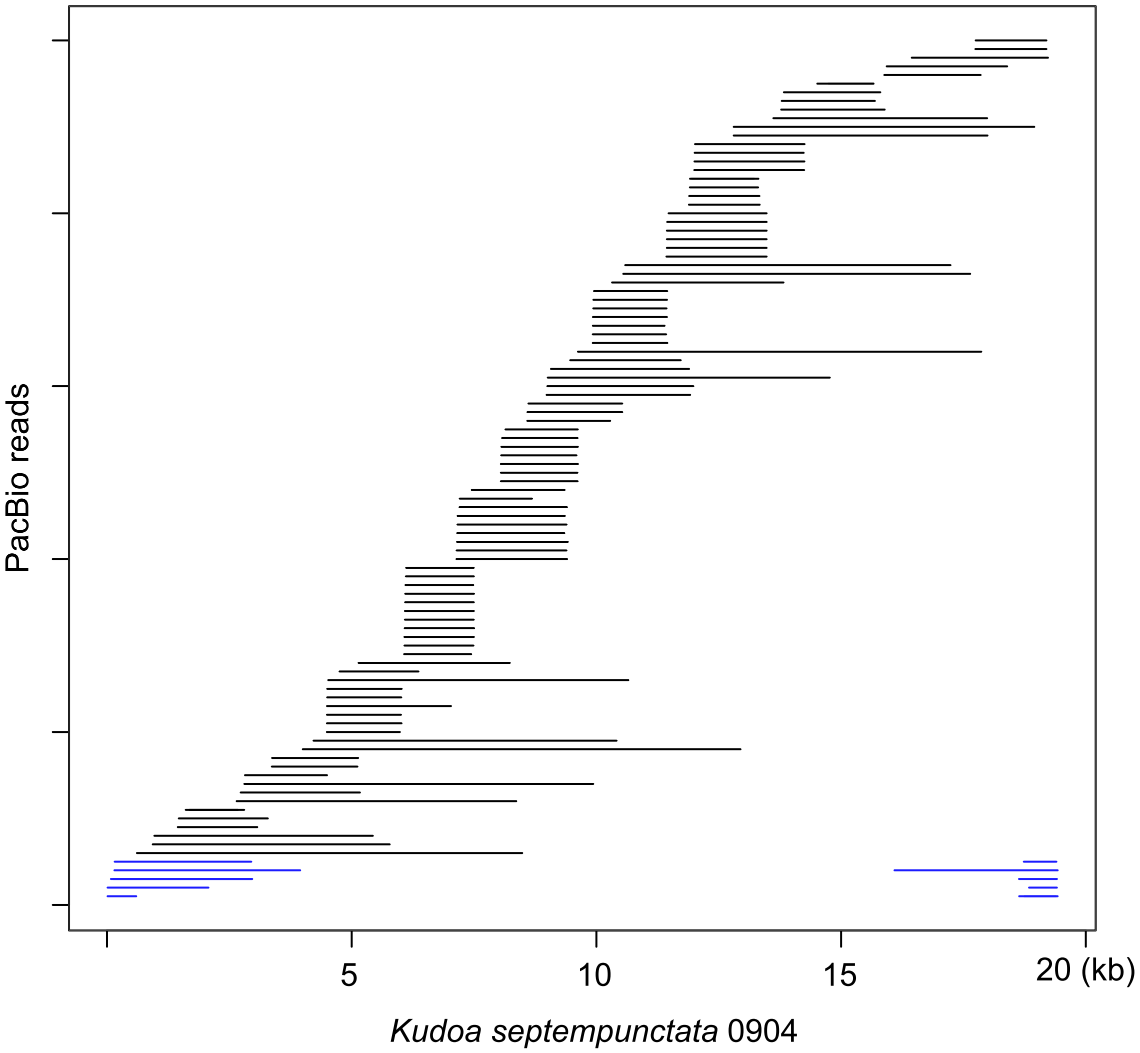

Supplement: S2 Fig — (PDF) [file pone.0132030.s002.pdf]
