# Supplementary material for: The Mitochondrial Genomes of a Myxozoan Genus Kudoa Are Extremely Divergent in Metazoa
Source: PLoS One. 2015 Jul 6;10(7):e0132030. doi: 10.1371/journal.pone.0132030 (PMC4492933; doi:10.1371/journal.pone.0132030)

S3 Fig.

(*A*) Genes annotated on the mitochondrial genome of *K. septempunctata* isolate 0904. (*B*) RNA reads mapped to the genome. The horizontal axis represents the position on genome sequence, and the vertical axis represents the depth of the mapped reads. The annotation of the small and large subunit rRNA genes (*rns* and *rnl*) is supported by the abundant amount of expressed RNA. The GenomeJack software was used for the plotting.

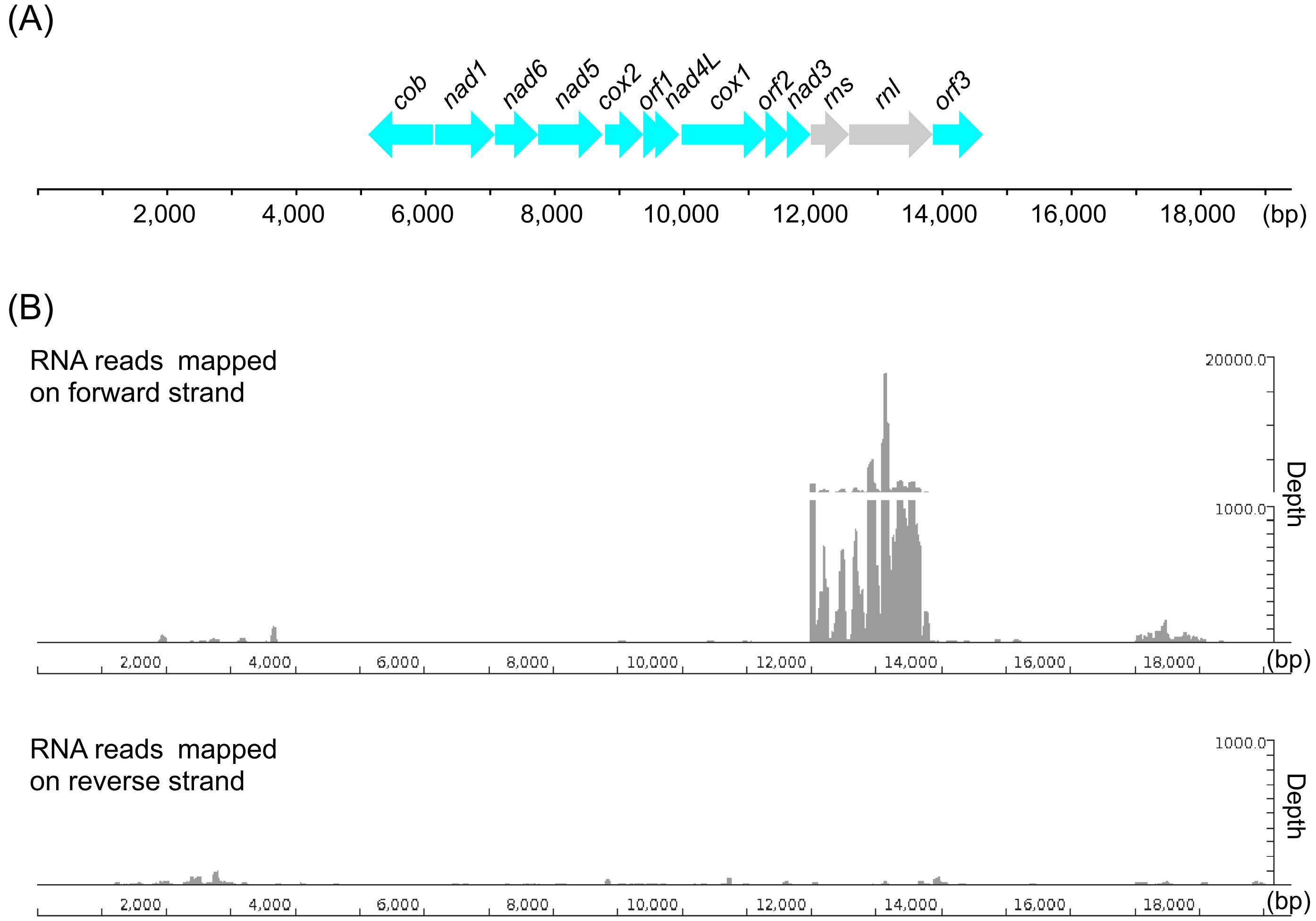

Supplement: S3 Fig — (PDF) [file pone.0132030.s003.pdf]
