# Supplementary material for: The Mitochondrial Genomes of a Myxozoan Genus Kudoa Are Extremely Divergent in Metazoa
Source: PLoS One. 2015 Jul 6;10(7):e0132030. doi: 10.1371/journal.pone.0132030 (PMC4492933; doi:10.1371/journal.pone.0132030)

S4 Fig.

Mitochondrial aerobic respiration observed in *K. septempunctata* myxospores. Multiple sections of confocal microscope images are shown. Relative position of the sectional images in the z-axis is denoted above; z = 0 μm was shown in Fig. 5. Mitochondria are stained red with Rhodamine 123, and nuclei are stained blue with Hoechst 33258. The myxospore in top panels appears in transverse section; one nucleus in sporoplasm is visible at z = 3 μm. The myxospore in the lower half of bottom panels appears in radial section; three nuclei in sporoplasm are visible at z = 1, 3, 4 μm.

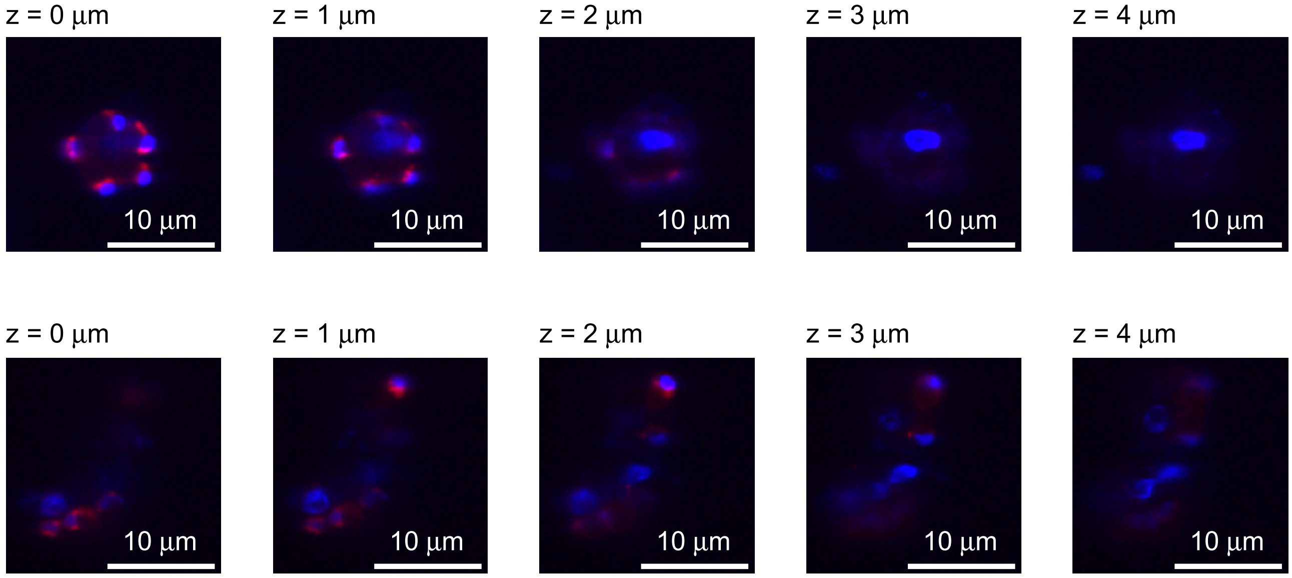

Supplement: S4 Fig — (PDF) [file pone.0132030.s004.pdf]
