# Supplementary material for: The Mitochondrial Genomes of a Myxozoan Genus Kudoa Are Extremely Divergent in Metazoa
Source: PLoS One. 2015 Jul 6;10(7):e0132030. doi: 10.1371/journal.pone.0132030 (PMC4492933; doi:10.1371/journal.pone.0132030)

S5 Fig.

DNA sequencing depth of the mitochondrial chromosome of *K. septempunctata* isolate 0904. The length (horizontal axis) and depth (vertical axis) is plotted for the DNA contigs assembled from Illumina reads. To selectively assemble the mitochondrial genome, we restricted the assembly to 85-mers of depth >2000. The contig marked 'mt' has depth ~27,000 and length ~19,000 bp, and represents the mitochondrial chromosome. Since the contigs with similar depth are small (<300 bp), it is unlikely that other mitochondrial chromosomes exist, unless very small in size or low in copy number.

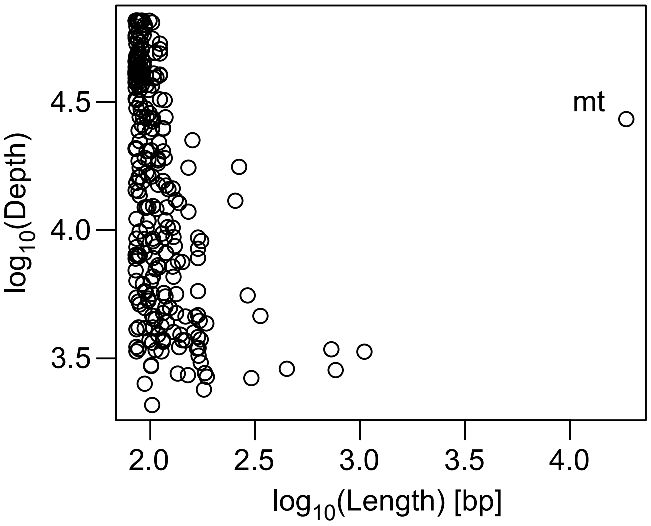

Supplement: S5 Fig — (PDF) [file pone.0132030.s005.pdf]
