# Supplementary material for: The Mitochondrial Genomes of a Myxozoan Genus Kudoa Are Extremely Divergent in Metazoa
Source: PLoS One. 2015 Jul 6;10(7):e0132030. doi: 10.1371/journal.pone.0132030 (PMC4492933; doi:10.1371/journal.pone.0132030)

S6 Fig.

Transfer RNA genes discovered in the mitochondrial genomes of *Kudoa* species. For each tRNA, the location in genome is indicated by a range of base pair position. No tRNA genes were discovered for *K. septempunctata* 0904 and *K. iwatai*.

Kudoa septempunctata isolate 210204

3979..4051
trnV-uac, Cove=7.87

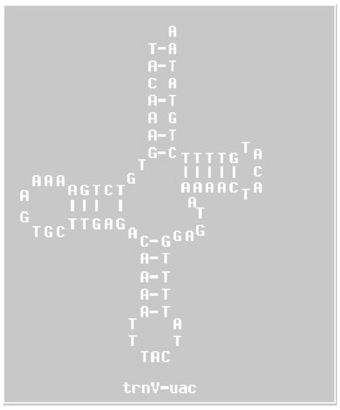

Kudoa hexapunctata

revcomp(330..396)
trnG-ucc, Cove=17.01

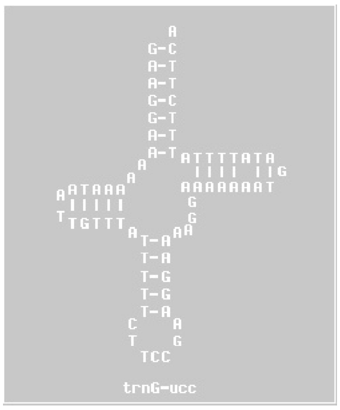

17568..17656
trnG-ccc, Cove=12.53

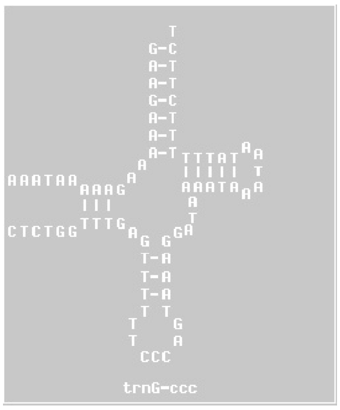

revcomp(17692..17757)
trnV-aac, Cove=12.88

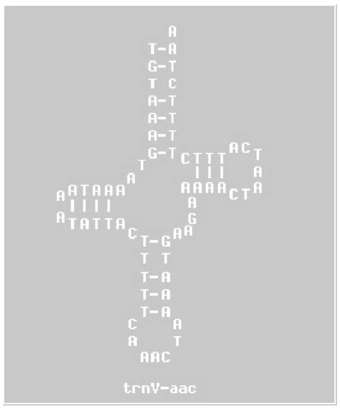

revcomp(18027..18113)
trnW-cca, Cove=10.40

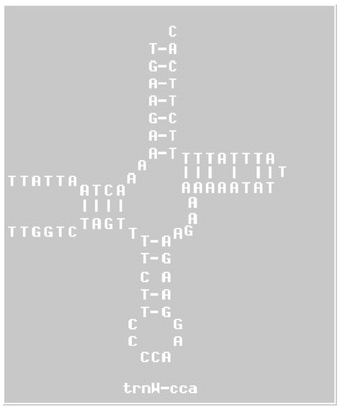

Supplement: S6 Fig — (PDF) [file pone.0132030.s006.pdf]
