# Supplementary material for: The Mitochondrial Genomes of a Myxozoan Genus Kudoa Are Extremely Divergent in Metazoa
Source: PLoS One. 2015 Jul 6;10(7):e0132030. doi: 10.1371/journal.pone.0132030 (PMC4492933; doi:10.1371/journal.pone.0132030)
Supplement: S1 Table — (PDF) [file pone.0132030.s007.pdf]

S1 Table. Mitochondrial metabolism genes expressed in *K. septempunctata* isolate 201204

Pathway	Gene name[a]	KEGG orthology ID	Expressed gene[b] ID	Length (nt)
Citrate cycle				
	CS, gltA	K01647	comp19138_c0_seq1	1413
	ACO, acnA	K01681	comp18554_c0_seq1	2456
	IDH1, IDH2, icd	K00031	comp18340_c0_seq2	1556
	IDH3	K00030	comp19417_c0_seq2	1178
	OGDH, sucA	K00164	comp21990_c0_seq1	309
	DLST, sucB	K00658	comp19912_c0_seq1	1603
	LSC1	K01899	comp6928_c0_seq1	242
	LSC2	K01900	comp18648_c0_seq1	1296
	SDHA, SDH1	K00234	comp19326_c0_seq1	2048
	SDHB, SDH2	K00235	comp19332_c0_seq1	954
	fumC	K01679	comp17430_c0_seq1	1680
	MDH1	K00025	comp16759_c0_seq1	1346
	MDH2	K00026	comp19943_c0_seq3	1776
Oxidative phosphorylation, complex I: NADH dehydrogenase				
	ND1			
	ND2			
	ND3			
	ND4			
	ND4L			
	ND5			
	ND6			
	Ndufs1	K03934	comp19612_c0_seq4	2366
	Ndufs2	K03935	comp20767_c0_seq2	1703
	Ndufs3	K03936	comp19779_c0_seq2	704
	Ndufs4	K03937	comp20017_c0_seq2	824
	Ndufs5			
	Ndufs6	K03939	comp18755_c0_seq1	575
	Ndufs7	K03940	comp19529_c0_seq1	708
	Ndufs8	K03941	comp16079_c0_seq1	647
	Ndufv1	K03942	comp21282_c2_seq1	1474
	Ndufv2	K03943	comp19671_c0_seq1	733
	Ndufa2	K03946	comp13859_c0_seq1	437
	Ndufa4			
	Ndufa5	K03949	comp17672_c0_seq1	460
	Ndufa6			
	Ndufa7			
	Ndufa8			
	Ndufa9	K03953	comp20738_c0_seq1	1373
	Ndufa10			
	Ndufab1			
	Ndufa11			
	Ndufa12	K11352	comp8404_c0_seq1	341
	Ndufa13			
	Ndufb2			
	Ndufb3			
	Ndufb7			
	Ndufb8			
	Ndufb9			
	Ndufb10			

Oxidative phosphorylation, complex II: Succinate dehydrogenase / Fumarate reductase			
SDHC			
SDHD			
SDHA	K00234	comp19326_c0_seq1	2048
SDHB	K00235	comp19332_c0_seq1	954
Oxidative phosphorylation, complex III: Cytochrome c reductase			
UQCRFS1	K00411	comp19439_c0_seq1	964
Cytb			
Cyt1	K00413	comp7739_c0_seq1	946
QCR2			
QCR6			
QCR7			
QCR8			
QCR9			
QCR10			
Oxidative phosphorylation, complex IV: Cytochrome c oxidase			
COX10	K02257	comp21127_c0_seq1	1242
COX3			
COX1	K02256	comp13813_c0_seq1	238
COX2	K02261		
COX4			
COX5A			
COX5B			
COX6A			
COX6B			
COX7A			
COX7C			
COX11	K02258	comp19547_c0_seq2	795
COX15	K02259	comp20983_c2_seq3	567
COX17	K02260	comp63280_c0_seq1	241
Oxidative phosphorylation, complex V: F-type ATPase			
ATPeF1A	K02132	comp21000_c0_seq1	1822
ATPeF1B	K02133	comp61772_c0_seq1	464
ATPeF1G	K02136	comp77451_c0_seq1	701
ATPeF1D	K02134	comp15686_c0_seq1	502
ATPeF1E			
ATPeF0O	K02137	comp17561_c0_seq1	660
ATPeF0B			
ATPeF0C	K02128	comp12825_c0_seq1	490
ATPeF0D			
ATPeF0E (ATP6)	K02126		
ATPeFG			
ATPeF0F6			
ATPeF08 (ATP8)	K02125		

KEGG gene orthology are listed if present in *Hydra vulgaris* or *Nematostella vectensis.*

[a] Genes undetected in *K. septempunctata* transcriptome are in gray font.

[b] When there were multiple hits, the longest transcript was chosen.
