# Supplementary material for: The Mitochondrial Genomes of a Myxozoan Genus Kudoa Are Extremely Divergent in Metazoa
Source: PLoS One. 2015 Jul 6;10(7):e0132030. doi: 10.1371/journal.pone.0132030 (PMC4492933; doi:10.1371/journal.pone.0132030)
Supplement: S2 Table — (PDF) [file pone.0132030.s008.pdf]

**S2 Table. DNA and RNA library preparation and sequencing**

**A. DNA libraries**

| Species, isolate | Library ID | Type[a] | Insertion size (bp) | Number of pairs (or reads for PacBio) | Read 1 length (bp) | Read 2 length (bp) | Sequencer |
|---|---|---|---|---|---|---|---|
| *Kudoa septempunctata*, 0904 | 300bpnomerge | PE | 200 | 51,219,620 | 125 | 125 | GAIIx |
| | 1kb | PE | 750 | 37,142,657 | 125 | 125 | GAIIx |
| | PacBio | PacBio | ~10kb | 171,789 | ~10kb | – | RSII |
| *Kudoa septempunctata*, 201204 | 750-1000bp | PE | 683 | 11,198,350 | 300 | 200 | MiSeq |
| | 1000-1500bp | PE | 1042 | 24,819,900 | 81 | 81 | GAIIx |
| | 1000-1500bp2 | PE | 1042 | 81,861,771 | 81 | 81 | GAIIx |
| | 1000-1500bp3 | PE | 1042 | 85,229,517 | 81 | 81 | GAIIx |
| | 1500-2000bp | PE | 1491 | 7,916,881 | 81 | 81 | GAIIx |
| | 1500-2000bp2 | PE | 1491 | 68,359,906 | 81 | 81 | GAIIx |
| *Kudoa hexapunctata*, 2012.6.3 | neothunni_Nextera | PE | 1355 | 22,072,249 | 81 | 81 | GAIIx |
| *Kudoa iwatai*, KI-001 | iwatai_mt | PE | 650 | 29,199 | 300 | 300 | MiSeq |
| | iwatai_wg | PE | 600 | 3,432,679 | 300 | 300 | MiSeq |

**B. RNA libraries**

| Species, isolate | Library ID | Type[a] | Insertion size | Number of pairs | Read 1 length (bp) | Read 2 length (bp) | Sequencer |
|---|---|---|---|---|---|---|---|
| *Kudoa septempunctata*, 0904 | long PE | PE | 150 | 7,070,734 | 81 | 81 | GAIIx |
| *Kudoa septempunctata*, 201204 | Kudoa-0h | PE | 233 | 16,273,130 | 81 | 81 | GAIIx |

[a] PE indicates paired end library for Illumina seqeuncers
