# Supplementary material for: The Mitochondrial Genomes of a Myxozoan Genus Kudoa Are Extremely Divergent in Metazoa
Source: PLoS One. 2015 Jul 6;10(7):e0132030. doi: 10.1371/journal.pone.0132030 (PMC4492933; doi:10.1371/journal.pone.0132030)
Supplement: S3 Table — (PDF) [file pone.0132030.s009.pdf]

S3 Table. Primers used to confirm the mitochondrial genome assemblies

Primer			*Kudoa septempunctata* 0904 AB731753			*Kudoa septempunctata* 201204 LC009436		
No	Sequence	Length (nt)	Start	End	Strand	Start	End	Strand
1	GTGATTTTGTAGAGTGCGAAC	21	1055	1035	R	830	810	R
2	GTTCGCACTCTACAAAATCAC	21	1035	1055	F	810	830	F
3	TTGTTGTCAGCTAGGAGAGAG	21	1432	1412	R	1207	1187	R
4	AAATTGCGACACATTACTTTC	21	3220	3240	F			
5	GCTCCTTTAGCTAGGAACACC	21	3806	3786	R			
6	AGAAGAAGCAAGCCTAACAG	20				5175	5156	R
7	CAATCATTTGCGTTCATGTC	20	5435	5454	F	5404	5423	F
8	GATACACAGACTCTCCTGCTG	21	5733	5753	F	5702	5722	F
9	ATCAGGTTGCGTCCTATATT	20	5872	5853	R	5841	5822	R
10	CACAAAGCTTAAAACCATCAC	21	6301	6281	R	6270	6250	R
11	CTCACCAGGCAAATAACAAG	20	7177	7158	R	7146	7127	R
12	TTTAGTGCTTTGTTTAGCACC	21	8025	8045	F	7994	8014	F
13	GTCCAAATCTTACCCAAAGAG	21	8724	8704	R	8693	8673	R
14	CCTTAATTGGTGATTTCCTG	20	10194	10213	F	10163	10182	F
15	GGAATTTTGCTTGATAGAACA	21	10570	10590	F	10539	10559	F
16	TATGGCAAAGAAGGTCTGAT	20	10741	10760	F	10710	10729	F
17	AAGGAAGGAAAATAGTCATGG	21	11194	11174	R	11163	11143	R
18	CCATGACTATTTTCCTTCCTT	21	11174	11194	F	11143	11163	F
19	TCTAGGGATTCCACAAAGAC	20	11226	11207	R	11195	11176	R
20	GTTCCAACAAGTCCATGAAA	20	12690	12709	F	12659	12678	F
21	GACTTTATGGACAACTCAGC	20	13178	13159	R	13147	13128	R
22	TACTGGCAGCAGTATCTTGAC	21	13287	13307	F	13256	13276	F
23	CGTTTTAAACTCAGATCATGC	21	13714	13694	R	13683	13663	R
24	GCATGATCTGAGTTTAAAACG	21	13694	13714	F	13663	13683	F
25	CACCTGTAGGGCAAAATCTA	20	15491	15472	R	15461	15442	R
26	TAGATTTTGCCCTACAGGTG	20	15472	15491	F	15442	15461	F
27	ATCCTCTTTTTCTACTTTCC	20	15578	15597	F	15548	15567	F
28	AACGAAAAAGGACTCATATCC	21	16079	16099	F	16048	16068	F
29	ACCCCTCCAATTATAAACCG	20				16087	16068	R
30	AACGGATATGAGTCCTTTTTC	21	16102	16082	R			
31	AGCCCCTCCAATCATAAAAC	20	16119	16100	R			
32	CCGTAGAGAGTGGTAGATCG	20	17170	17189	F	17139	17158	F
33	CTCACAAGGGGAAGAATAGT	20	17760	17741	R	17729	17710	R
34	AGAGCAGGAGGTTAGAATTT	20	18244	18263	F	18212	18231	F
35	CAGAGTGGTACAGTCCATTTC	21	18793	18813	F	18760	18780	F
36	CGAGAAATGGACTGTACCAC	20	18816	18797	R	18783	18764	R
37	GGCAAATTCTTACACCCCTT	20	18856	18875	F	18823	18842	F

Primer			*Kudoa iwatai* LC009438		
No	Sequence	Length (nt)	Start	End	Strand
38	ATTTTCTCAAAGGAGTCGGG	20	1599	1580	R
39	AAAGGTAACACCAGCTAGAC	20	2005	2024	F
40	AGCATGCCTATAAACTACAG	20	3568	3549	R
41	GGATATTAGCTGGAAAGGAT	20	5964	5945	R
42	TGATGTTGAGGTATATCTGG	20	7277	7296	F
43	GCCATAACCATACCTTACTA	20	8196	8215	F
			8384	8365	R
44	CAGGACATGTTACTTTGGAT	20	9787	9806	F
45	CCTATCTATTTCAGGAACCA	20	10866	10847	R
46	TGGTTATCCTTCCATCATTC	20	11473	11454	R
47	AGTGCTAATGCTAATGATGG	20	11590	11609	F
48	GATTGAACTTGTGGGAAACT	20	12046	12065	F
49	GTGACATGGCTGAATATAAG	20	13177	13196	F
50	GGAACCAGTACAAGAAGAAA	20	13639	13620	R
51	CAGGATTAGGTATAGGATGA	20	14852	14871	F
