# Supplementary material for: The Mitochondrial Genomes of a Myxozoan Genus Kudoa Are Extremely Divergent in Metazoa
Source: PLoS One. 2015 Jul 6;10(7):e0132030. doi: 10.1371/journal.pone.0132030 (PMC4492933; doi:10.1371/journal.pone.0132030)
Supplement: S4 Table — (PDF) [file pone.0132030.s010.pdf]

S4 Table. Mitochondrial genomes used for phylogenetic analyses

Group	Phylum	Class	Species	NCBI accession no. of mitochondrial genome
Fungi (outgroup)			*Rhizopus oryzae*	NC_006836.1
Choanoflagellida (outgroup)			*Monosiga brevicollis*	NC_004309.1
	Placozoa		*Trichoplax adhaerens*	NC_008151.2
	Placozoa		*Placozoan sp. BZ10101*	NC_008832.2
	Placozoa		*Placozoan sp. BZ49*	NC_008833.2
	Placozoa		*Placozoan sp. BZ2423*	NC_008834.2
	Porifera	Demospongia	*Corticium candelabrum*	NC_014872.1
	Porifera	Demospongia	*Oscarella carmela*	NC_009090.1
	Porifera	Demospongia	*Plakina monolopha*	NC_014884.1
	Porifera	Demospongia	*Plakinastrella cf. onkodes DVL-2011*	NC_010217.1
	Porifera	Demospongia	*Igernella notabilis*	NC_010216.1
	Porifera	Demospongia	*Ircinia strobilina*	NC_013662.1
	Porifera	Demospongia	*Aplysina fulva*	NC_010203.1
	Porifera	Demospongia	*Chondrilla aff. nucula CHOND*	NC_010208.1
	Porifera	Demospongia	*Halisarca dujardini*	NC_010212.1
	Porifera	Demospongia	*Amphimedon compressa*	NC_010201.1
	Porifera	Demospongia	*Amphimedon queenslandica*	NC_008944.1
	Porifera	Demospongia	*Callyspongia plicifera*	NC_010206.1
	Porifera	Demospongia	*Xestospongia muta*	NC_010211.1
	Porifera	Demospongia	*Agelas schmidti*	NC_010213.1
	Porifera	Demospongia	*Axinella corrugata*	NC_006894.1
	Porifera	Demospongia	*Cinachyrella kuekenthali*	NC_010198.1
	Porifera	Demospongia	*Ephydatia muelleri*	NC_010202.1
	Porifera	Demospongia	*Geodia neptuni*	NC_006990.1
	Porifera	Demospongia	*Lubomirskia baicalensis*	NC_013760.1
	Porifera	Demospongia	*Suberites domuncula*	NC_010496.1
	Porifera	Demospongia	*Tethya actinia*	NC_006991.1
	Porifera	Demospongia	*Topsentia ophiraphidites*	NC_010204.1
	Porifera	Hexactinellida	*Aphrocallistes vastus*	NC_010769.1
	Cnidaria	Myxozoa	*Kudoa septempunctata* 0904	AB731753
	Cnidaria	Myxozoa	*Kudoa septempunctata* 201204	LC009436
	Cnidaria	Myxozoa	*Kudoa hexapunctata*	LC009437
	Cnidaria	Myxozoa	*Kudoa iwatai*	LC009438
	Cnidaria	Hexacorallia	*Metridium senile*	NC_000933.1
	Cnidaria	Hexacorallia	*Nematostella sp. JVK-2006*	NC_008164.1
	Cnidaria	Hexacorallia	*Chrysopathes formosa*	NC_008411.1
	Cnidaria	Hexacorallia	*Stichopathes lutkeni (synonym: Cirrhipathes lutkeni)*	NC_018377.1
	Cnidaria	Hexacorallia	*Leiopathes glaberrima*	FJ597643 FJ597644
	Cnidaria	Hexacorallia	*Discosoma sp. CASIZ 168915*	NC_008071.1
	Cnidaria	Hexacorallia	*Rhodactis sp. CASIZ 171755*	NC_008158.1
	Cnidaria	Hexacorallia	*Ricordea florida*	NC_008159.1
	Cnidaria	Hexacorallia	*Acropora tenuis*	NC_003522.1
	Cnidaria	Hexacorallia	*Agaricia humilis*	NC_008160.1
	Cnidaria	Hexacorallia	*Anacropora matthai*	NC_006898.1
	Cnidaria	Hexacorallia	*Astrangia sp. JVK-2006*	NC_008161.1
	Cnidaria	Hexacorallia	*Colpophyllia natans*	NC_008162.1
	Cnidaria	Hexacorallia	*Lophelia pertusa*	NC_015143.1
	Cnidaria	Hexacorallia	*Madracis mirabilis (synonym: Madracis myriaster)*	NC_011160.1
	Cnidaria	Hexacorallia	*Montastraea annularis*	NC_007224.1
	Cnidaria	Hexacorallia	*Montastraea faveolata*	NC_007226.1
	Cnidaria	Hexacorallia	*Montastraea franksi*	NC_007225.1
	Cnidaria	Hexacorallia	*Montipora cactus*	NC_006902.1
	Cnidaria	Hexacorallia	*Mussa angulosa*	NC_008163.1
	Cnidaria	Hexacorallia	*Pavona clavus*	NC_008165.1
	Cnidaria	Hexacorallia	*Pocillopora damicornis*	NC_009797.1
	Cnidaria	Hexacorallia	*Pocillopora eydouxi*	NC_009798.1
	Cnidaria	Hexacorallia	*Porites porites*	NC_008166.1
	Cnidaria	Hexacorallia	*Seriatopora caliendrum*	NC_010245.2
	Cnidaria	Hexacorallia	*Seriatopora hystrix*	NC_010244.2
	Cnidaria	Hexacorallia	*Siderastrea radians*	NC_008167.1
	Cnidaria	Hexacorallia	*Stylophora pistillata*	NC_011162.1
	Cnidaria	Hexacorallia	*Savalia savaglia*	NC_008827.1
	Cnidaria	Hexacorallia	*Ceriantheopsis americanus*	JX023261-JX023265
	Cnidaria	Octocorallia	*Acanella eburnea*	NC_011016.2
	Cnidaria	Octocorallia	*Briareum asbestinum*	NC_008073.1
	Cnidaria	Octocorallia	*Corallium konojoi*	NC_015406.1
	Cnidaria	Octocorallia	*Dendronephthya castanea*	GU047877.1
	Cnidaria	Octocorallia	*Dendronephthya gigantea*	NC_013573.1
	Cnidaria	Octocorallia	*Dendronephthya mollis*	HQ694725.1
	Cnidaria	Octocorallia	*Dendronephthya putteri*	HQ694726.1
	Cnidaria	Octocorallia	*Dendronephthya suensoni*	GU047878.1
	Cnidaria	Octocorallia	*Echinogorgia complexa*	HQ694727.1
	Cnidaria	Octocorallia	*Euplexaura crassa*	HQ694728.1

	Cnidaria	Octocorallia *Keratoisidinae sp. BAL208-1*	NC_010764.1
	Cnidaria	Octocorallia *Paracorallium japonicum*	NC_015405.1
	Cnidaria	Octocorallia *Pseudopterogorgia bipinnata*	NC_008157.1
	Cnidaria	Octocorallia *Sarcophyton glaucum*	AF064823 AF063191
	Cnidaria	Octocorallia *Scleronephthya gracillimum*	GU047879.1
	Cnidaria	Octocorallia *Sinularia peculiaris*	NC_018379.1
	Cnidaria	Octocorallia *Renilla muelleri*	NC_018378.1
	Cnidaria	Octocorallia *Stylatula elongata*	NC_018380.1
	Cnidaria	Cubozoa *Chironex fleckeri*	JN700963-JN700968
	Cnidaria	Cubozoa *Chiropsalmus quadrumanus*	JN700969-JN700974
	Cnidaria	Hydrozoa *Cubaia aphrodite*	JN700942.1
	Cnidaria	Hydrozoa *Ectopleura larynx*	JN700938.1
	Cnidaria	Hydrozoa *Hydra magnipapillata*	NC_011220.1 NC_011221.1
	Cnidaria	Hydrozoa *Hydra oligactis*	NC_010214.1
	Cnidaria	Hydrozoa *Hydra vulgaris*	BN001179 BN001180
	Cnidaria	Hydrozoa *Millepora sp. EK-2011*	JN700943.1
	Cnidaria	Hydrozoa *Pennaria disticha (synonym: Pennaria tiarella)*	JN700950.1
	Cnidaria	Hydrozoa *Clava multicornis*	JN700935.1
	Cnidaria	Hydrozoa *Nemopsis bachei*	JN700947.1
	Cnidaria	Hydrozoa *Laomedea flexuosa*	JN700945.1
	Cnidaria	Coronatae *Linuche unguiculata*	JN700939.1
	Cnidaria	Discomedusae *Cassiopea andromeda*	JN700934.1
	Cnidaria	Discomedusae *Cassiopea frondosa*	JN700936.1
	Cnidaria	Discomedusae *Catostylus mosaicus*	JN700940.1
	Cnidaria	Discomedusae *Aurelia aurita*	NC_008446.1
	Cnidaria	Discomedusae *Chrysaora quinquecirrha*	HQ694730.1
	Cnidaria	Discomedusae *Chrysaora sp. EK-2011*	JN700941.1
	Cnidaria	Discomedusae *Cyanea capillata*	JN700937.1
	Cnidaria	Discomedusae *Pelagia noctiluca*	JN700949.1
	Cnidaria	Staurozoa *Craterolophus convolvulus*	JN700975 JN700976
	Cnidaria	Staurozoa *Haliclystus sanjuanensis*	JN700944.1
	Cnidaria	Staurozoa *Lucernaria janetae*	JN700946.1
	Ctenophora	*Mnemiopsis leidyi*	NC_016117.1
	Ctenophora	*Pleurobrachia bachei*	NC_016697.1
Bilateria; Deuterostomia	Chordata	*Homo sapiens*	NC_011137.1
Bilateria; Deuterostomia	Chordata	*Paralichthys olivaceus*	NC_002386.1
Bilateria; Deuterostomia	Chordata	*Danio rerio*	NC_002333.2
Bilateria; Deuterostomia	Chordata	*Trioceros melleri*	NC_014176.1
Bilateria; Deuterostomia	Echinodermata	*Strongylocentrotus pallidus*	NC_009941.1
Bilateria; Deuterostomia	Echinodermata	*Neogymnocrinus richeri*	NC_007689.1
Bilateria; Deuterostomia	Hemichordata	*Saccoglossus kowalevskii*	NC_007438.1
Bilateria; Deuterostomia	Xenoturbellida	*Xenoturbella bocki*	NC_008556.1
Bilateria; Lophotrochozoa	Platyhelminthes	*Taenia saginata*	NC_009938.1
Bilateria; Lophotrochozoa	Platyhelminthes	*Gyrodactylus salaris*	NC_008815.1
Bilateria; Lophotrochozoa	Platyhelminthes	*Microcotyle sebastis*	NC_009055.1
Bilateria; Lophotrochozoa	Platyhelminthes	*Schistosoma mansoni*	NC_002545.1
Bilateria; Lophotrochozoa	Platyhelminthes	*Clonorchis sinensis*	NC_012147.2
Bilateria; Lophotrochozoa	Platyhelminthes	*Dugesia ryukyuensis*	AB618488.1
Bilateria; Lophotrochozoa	Platyhelminthes	*Dugesia japonica*	NC_016439.1
Bilateria; Lophotrochozoa	Chaetognatha	*Zonosagitta nagae*	NC_013810.1
Bilateria; Lophotrochozoa	Chaetognatha	*Spadella cephaloptera*	NC_006386.1
Bilateria; Lophotrochozoa	Annelida	*Terebellides stroemii*	NC_011014.1
Bilateria; Lophotrochozoa	Echiura	*Urechis caupo*	NC_006379.1
Bilateria; Lophotrochozoa	Brachiopoda	*Terebratulina retusa*	NC_000941.1
Bilateria; Lophotrochozoa	Bryozoa	*Flustrellidra hispida*	NC_008192.1
Bilateria; Lophotrochozoa	Bryozoa	*Tubulipora flabellaris*	NC_015646.1
Bilateria; Lophotrochozoa	Entoprocta	*Loxocorone allax*	NC_010431.1
Bilateria; Lophotrochozoa	Mollusca	*Chaetoderma nitidulum*	NC_013846.1
Bilateria; Lophotrochozoa	Mollusca	*Lampsilis ornata*	NC_005335.1
Bilateria; Lophotrochozoa	Mollusca	*Loligo bleekeri*	NC_002507.1
Bilateria; Lophotrochozoa	Mollusca	*Aplysia californica*	NC_005827.1
Bilateria; Lophotrochozoa	Mollusca	*Katharina tunicata*	NC_001636.1
Bilateria; Lophotrochozoa	Mollusca	*Graptacme eborea*	NC_006162.1
Bilateria; Lophotrochozoa	Nemertea	*Cephalothrix simula*	NC_012821.1
Bilateria; Lophotrochozoa	Sipuncula	*Sipunculus nudus*	NC_011826.1
Bilateria; Lophotrochozoa	Acanthocephala	*Oncicola luehei*	NC_016754.1
Bilateria; Lophotrochozoa	Acanthocephala	*Leptorhynchoides thecatus*	NC_006892.1
Bilateria; Lophotrochozoa	Rotifera	*Rotaria rotatoria*	NC_013568.1
Bilateria; Lophotrochozoa	Rotifera	*Brachionus plicatilis*	NC_010472.1 NC_010484.1
Bilateria; Ecdysozoa	Arthropoda	*Drosophila melanogaster*	NC_001709.1
Bilateria; Ecdysozoa	Arthropoda	*Limulus polyphemus*	NC_003057.1
Bilateria; Ecdysozoa	Onychophola	*Epiperipatus biolleyi*	NC_009082.1
Bilateria; Ecdysozoa	Tardigrada	*Hypsibius dujardini*	NC_014848.1
Bilateria; Ecdysozoa	Priapulida	*Priapulus caudatus*	NC_008557.1
Bilateria; Ecdysozoa	Nematoda	*Anisakis simplex*	NC_007934.1
Bilateria; Ecdysozoa	Nematoda	*Pristionchus pacificus*	NC_015245.1

Bilateria; Ecdysozoa	Nematoda	*Enterobius vermicularis*	NC_011300.1
Bilateria; Ecdysozoa	Nematoda	*Caenorhabditis elegans*	NC_001328.1
Bilateria; Ecdysozoa	Nematoda	*Brugia malayi*	NC_004298.1
Bilateria; Ecdysozoa	Nematoda	*Radopholus similis*	NC_013253.1
Bilateria; Ecdysozoa	Nematoda	*Trichinella spiralis*	NC_002681.1
Basal bilateria[a]	Acoelomorpha	*Symsagittifera roscoffensis*	NC_014578.1

[a] Debated between basal Bilateria [Mwinyi et al. BMC Evolutionary Biology 2010, 10:309] and basal Deuterostomia [Philippe et al. Nature 2011, 470:255].
